# Presynaptic supervision of cortical spine dynamics in motor learning

**DOI:** 10.1126/sciadv.abm0531

**Published:** 2022-07-27

**Authors:** Jaerin Sohn, Mototaka Suzuki, Mohammed Youssef, Sayuri Hatada, Matthew E. Larkum, Yasuo Kawaguchi, Yoshiyuki Kubota

**Affiliations:** ^1^ Division of Cerebral Circuitry, National Institute for Physiological Sciences (NIPS), Okazaki 444-8787, Japan.; ^2^ Section of Electron Microscopy, Supportive Center for Brain Research, National Institute for Physiological Sciences (NIPS), Okazaki 444-8787, Japan.; ^3^ Neurocure Center for Excellence, Charité Universitätsmedizin Berlin, 10117 Berlin, Germany.; ^4^ Department of Animal Physiology, Faculty of Veterinary Medicine, South Valley University, Qena 83523, Egypt.; ^5^ Institute of Biology, Humboldt University of Berlin, 10117 Berlin, Germany.; ^6^ Department of Physiological Sciences, The Graduate University for Advanced Studies (SOKENDAI), Okazaki 444-8787, Japan.; ^7^ Brain Science Institute, Tamagawa University, Machida, Tokyo 194-8610, Japan.; ^8^ Support Unit for Electron Microscopy Techniques, Research Resources Division, RIKEN Center for Brain Science, Wako 351-0198, Japan.

## Abstract

In mammalian neocortex, learning triggers the formation and turnover of new postsynaptic spines on pyramidal cell dendrites. However, the biological principles of spine reorganization during learning remain elusive because the identity of their presynaptic neuronal partners is unknown. Here, we show that two presynaptic neural circuits supervise distinct programs of spine dynamics to execute learning. We imaged spine dynamics in motor cortex during learning and performed post hoc identification of their afferent presynaptic neurons. New spines that appeared during learning formed small transient contacts with corticocortical neurons that were eliminated on skill acquisition. In contrast, persistent spines with axons from thalamic neurons were formed and enlarged. These results suggest that pyramidal cell dendrites in motor cortex use a neural circuit division of labor during skill learning, with dynamic teaching contacts from top-down intracortical axons followed by synaptic memory formation driven by thalamic axons. Dual spine supervision may govern diverse skill learning in the neocortex.

## Teaser

Corticocortical and thalamocortical neural circuits presynaptically determine postsynaptic spine dynamics during learning.

## INTRODUCTION

Dendritic structural plasticity involving the formation and turnover of spines is a fundamental mechanism of the cellular basis of learning and memory in the mammalian neocortex. Spine dynamics observed during learning are a common feature of learning throughout the cortex, and common principles may operate across diverse forms of perception, action, and cognition. In mammalian model systems, motor learning is associated with the formation of new spines on apical tufts of layer 5 pyramidal cells in the primary motor cortex (M1) (*1*–*4*). Likewise, experimental shrinkage of dendritic spines in M1 that are potentiated during motor learning disrupts an acquired motor skill (*5*), indicating that spine plasticity during motor learning is required for skill acquisition. However, despite consensus evidence for a correlation between motor learning and postsynaptic spine plasticity (*1*, *2*, *6*, *7*), the presynaptic neuronal sources of synaptic inputs to new spines are largely unknown (*7*, *8*). This remains a major gap in knowledge because parameters of spine plasticity, including formation, elimination, and head size changes, may reflect the characteristics of presynaptic cell inputs. M1 neurons are postsynaptic targets for corticocortical (CC) networks and cortico-subcortical loops via the thalamus (*9*–*11*). Therefore, an understanding of the principles of network plasticity during learning will require a concurrent analysis of single spine dynamics and post hoc identification of presynaptic cells.

Numerous brain areas are mutually connected with M1 and may contribute to spine dynamics during motor learning. Concerted practice to master a new motor skill modifies ensemble neural activity in higher-order motor areas (*12*, *13*) and reorganizes communication between cortical and subcortical regions (*14*–*18*). Higher-order motor areas are responsible for goal-directed behavior and motor planning, supervising task-related feedback information for coordinated cortical activity (*12*, *19*, *20*). Subcortical efferents to the motor cortex are mediated by motor-related thalamic nuclei (*21*, *22*) and are associated with habitual or automatic movement control (*23*–*28*). CC and thalamocortical (TC) fibers abundantly terminate in layer 1 of M1 (*11*, *29*–*34*). In accord, we combined two-photon microscopy of spine dynamics, post hoc immunohistochemistry, and electron microscopy (EM) to search for the presynaptic neurons innervating individual new spines on the apical dendritic tufts in layer 1 formed during skill learning.

Previous studies have shown that, in the somatosensory cortex, new spines preferentially appear at the apical tuft of thick-tufted layer 5 pyramidal cells (*35*), presumably pontine-projecting cells [corticopontine (CPn) or pyramidal tract (PT) cells] (*11*), and that the new spines can have synaptic contacts (*35*, *36*). In the present study, we observed spine dynamics on the apical tuft of putative CPn cells in M1 and found that learning required the transient formation and elimination of spines innervated by CC axons, while new persistent spines from TC neurons were sustained and enlarged, indicating that dual neural circuits supervise spines for motor learning.

## RESULTS

### Spine formation correlates with motor skill acquisition

**Fig. 1.**
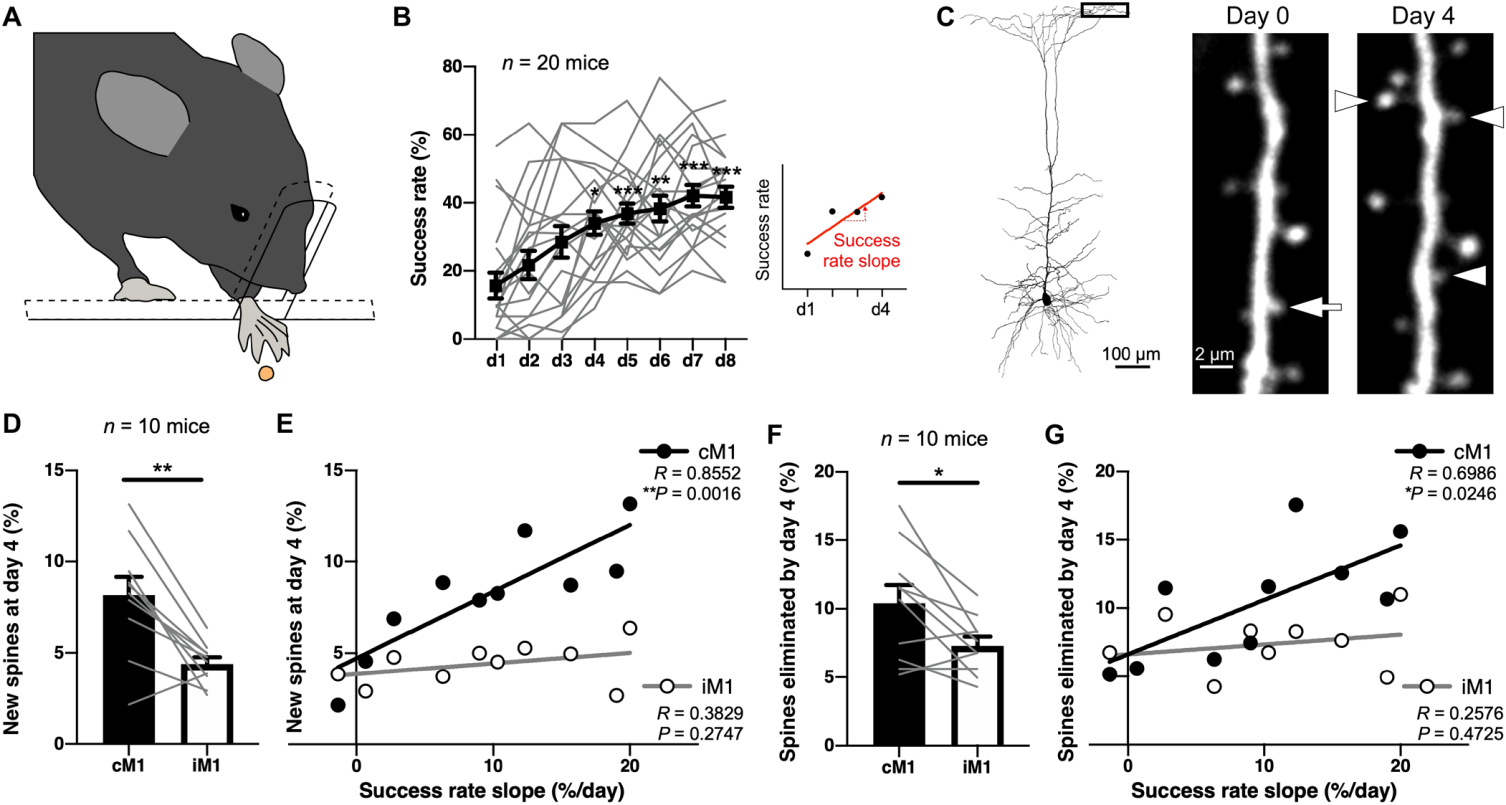
Motor skill improvement during the 4-day task training is correlated with spine formation on the apical tufts of layer 5 pyramidal cells in cM1. (**A**) Schematic diagram of the single-seed reaching task. (**B**) Success rate improvement over 8 days of forelimb training. Gray lines show success rate changes of individual mice, while a black line indicates means ± SEM. Success rate slope was the inclination of the linear regression over success rate values across 4 days for each animal. (**C**) Spines formed and eliminated during the 4-day motor learning. The apical tufts of layer 5 pyramidal cells within 40 μm from the pial surface were observed by in vivo two-photon microscopy. Arrowheads indicate newly formed spines, while an arrow indicates an eliminated spine. (**D**) Rates of newly formed spines found at day 4 of the training in cM1 and iM1 (means ± SEM). Gray lines show data of individual mice. (**E**) Correlation between success rate slopes and spine formation rates. (**F**) Rates of spines eliminated by day 4 of the training in cM1 and iM1 (means ± SEM). Gray lines show data of individual mice. (**G**) Correlations between success rate slopes and spine elimination rates of individual mice. *P < 0.05, **P < 0.01, and ***P < 0.001.

To characterize the spine dynamics in vivo during motor learning, we trained individual mice to reach for single seeds with their preferred forelimb that was determined beforehand (Fig. 1A and movie S1) (*1*). The mean rate of successful reaches increased during 8 days of practice (*n* = 20 mice; Fig. 1B). It was significantly higher at day 4 than at day 1, whereas there was no significant difference between mean rates at day 4 and at day 8 (*P* < 0.0001, Friedman test; day 1 versus day 4, *P* = 0.0312, day 4 versus day 8, *P* > 0.9999, Dunn’s multiple comparisons test; Fig. 1B). On average, mice could improve their skill during the initial 4 days of training.

We then observed spines on the apical tufts of pyramidal cell dendrites in vivo by two-photon imaging during 4 days of the training. In M1 of the Thy1-eGFP-M mouse line, a fraction of layer 5 pyramidal cells were labeled with green fluorescent protein (GFP), and 89.5 ± 2.4% (mean ± SEM; 260 of 289, *n* = 10 mice) of the cells expressed chicken ovalbumin upstream promoter transcription factor–interacting protein 2 (Ctip2), which is a marker of pontine-projecting cells (CPn or PT cells; fig. S1, A and B) (*30*, *37*). Ctip2-positive cells much more thickly extended their apical tufts (2.26 ± 0.24 mm per cell, mean ± SEM; total length per cell of the apical tuft within 40 μm from the pial surface) than Ctip2-negative cells (0.49 ± 0.13 mm per cell, mean ± SEM) (fig. S1, C and D). We therefore estimate that most (about 97.5%) of the GFP-labeled dendritic segments observable in vivo within a depth of 40 μm from the cortical surface are of CPn cells.

After individual training sessions, the apical tufts of layer 5 pyramidal cells were observed under a two-photon microscope within a depth of 40 μm from the cortical surface through bilaterally placed cranial windows on the forelimb area of M1 (*n* = 10 mice; Fig. 1C). Both the spine formation and elimination rates were significantly higher in M1 contralateral to the preferred forelimb (cM1) than the ipsilateral M1 (iM1) (*n* = 2247 spines in cM1 and 2194 spines in iM1, including stable, eliminated, and newly formed spines; spine formation rate, *W* = −51, *P* = 0.0059, two-tailed Wilcoxon matched-pairs signed-rank test; spine elimination rate, *W* = −39, *P* = 0.0195) (Fig. 1, D and F). The spine formation and elimination rates from days 0 to 4 in cM1 were linearly correlated with the increase in the success rate over the training sessions (where the increase in success rate was calculated as the slope of the linear regression over success rate values across 4 days for each animal as shown in Fig. 1B) (formation rate, *R* = 0.8552, *P* = 0.0016 in cM1 and *R* = 0.3829, *P* = 0.2747 in iM1, Pearson correlation coefficient; elimination rate, *R* = 0.6986, *P* = 0.0246 in cM1 and *R* = 0.2576, *P* = 0.4725 in iM1; Fig. 1, E and G), indicating that the spine dynamics underlie the skill improvement, consistent with previous studies (*1*–*5*).

### Improvement of the motor skill accompanies the formation of spines innervated by CC input

**Fig. 2.**
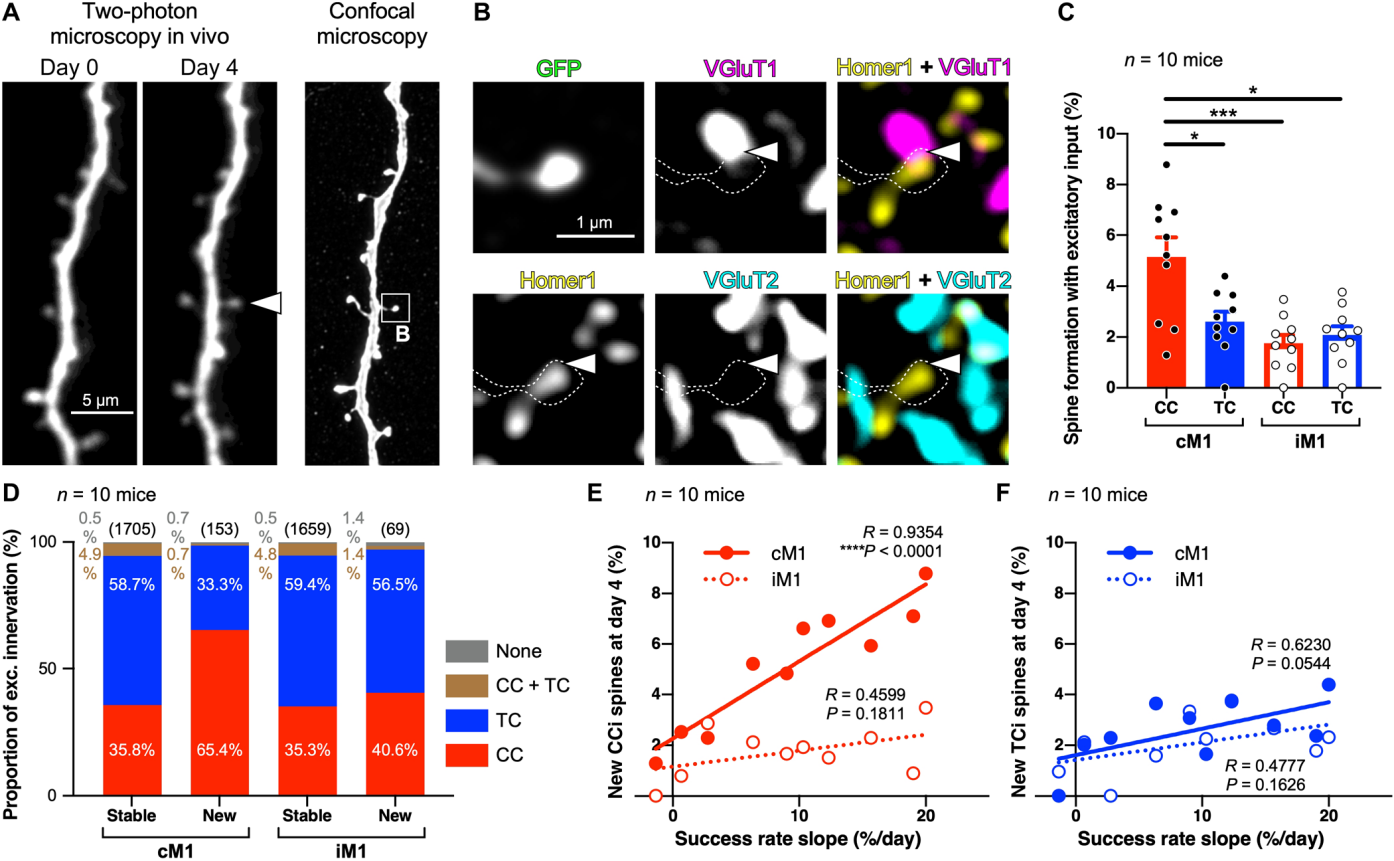
Motor skill improvement during the 4-day training accompanies CCi spine formation in cM1. (**A**) Left: Two-photon images in vivo at days 0 and 4. The arrowhead indicates a newly formed spine. Right: The dendrite image by confocal microscopy corresponding to the two-photon image. (**B**) Post hoc quadruple immunostaining for GFP, Homer1, VGluT1, and VGluT2. Dotted lines show the outline of the new spine in (A). Arrowheads indicate a putative synaptic input site. (**C**) Rates of CCi and TCi spine formations at day 4 in cM1 and iM1 (means ± SEM). (**D**) Proportion of excitatory innervations. A small number of spines seemed to receive both CC and TC inputs (CC + TC), and a further fewer number of spines were without any putative synaptic contact sites (none). Values in parentheses represent the number of spines analyzed. (**E** and **F**) Correlation between success rate slopes and spine formation rates with CC inputs (E) and TC inputs (F). *P < 0.05 and ***P < 0.001.

To characterize how learning-related spine formation depends on the input type (cortical or thalamic origin), we performed post hoc quadruple immunostaining for two presynaptic input markers as well as for a postsynaptic structure marker and GFP of postsynaptic dendrites with the spines observed in vivo (Fig. 2 and fig. S2). Instead of anterograde tracers that allow only partial visualization of presynaptic axon terminals, we adopted simultaneous immunostaining of almost all CC and TC axon terminals with their specific markers, vesicular glutamate transporters type I (VGluT1) and type II (VGluT2) as CC and TC excitatory presynaptic markers, respectively. The dendritic segments monitored in vivo under a two-photon microscope were successfully reidentified in the fixed brain sections by confocal microscopy (Fig. 2A and fig. S2, A and B). Then, we performed post hoc quadruple immunostaining for the presynaptic axon-type and postsynaptic structure markers in addition to GFP-labeled dendrites observed in vivo (Fig. 2B and fig. S2). The contact sites of these presynaptic puncta immunoreactive for VGluTs with the postsynaptic marker Homer1 in GFP-labeled spines were regarded as putative excitatory synaptic inputs (Fig. 2B and figs. S2, C and D, and S3) (*38*, *39*), confirmed by correlated light and electron microscopy (CLEM) that showed synaptic structure at the contact sites (fig. S4). VGluT puncta would contact Homer1 signals in GFP-labeled spine heads at a substantially lower rate by chance, as demonstrated by rotating the Homer1, VGluT1, and VGluT2 images by 90° (fig. S5). This is strong evidence that synapses were actually formed at the contact sites of these presynaptic and postsynaptic markers in the original images.

The post hoc immunohistochemistry revealed that the new spines in cM1 that formed by day 4 were about twofold more frequently innervated by CC axons than by TC ones (*P* = 0.0009, Friedman test; CC-cM1 versus TC-cM1, *P* = 0.0436; CC-cM1 versus CC-iM1, *P* = 0.0008; CC-cM1 versus TC-iM1, *P* = 0.0256; Dunn’s multiple comparisons test; *n* = 10 mice; Fig. 2C). While approximately ~60% of stable spines, i.e., spines observable throughout all imaging sessions, received inputs from TC axons (Fig. 2D), 65.4% of the new spines in cM1 were innervated by CC axons (Fig. 2D). In addition, the formation rate of corticocortically innervated (CCi) spines in cM1 was linearly correlated with the success rate increase of the motor task (*R* = 0.9354, *P* < 0.0001, Pearson correlation coefficient) and significantly different from that in iM1 [*R* = 0.4599, *P* = 0.1811; *P* = 0.0009, analysis of covariance (ANCOVA)–based regression comparison; Fig. 2E]. Although the formation rate of thalamocortically innervated (TCi) spines also had a trend to increase along with the success rate improvement (*R* = 0.6230, *P* = 0.0544, Pearson correlation coefficient), it was not significantly different from that of TCi spines in iM1 (*R* = 0.4778, *P* = 0.1626, Pearson correlation coefficient; *P* = 0.6063, ANCOVA) (Fig. 2F). These results show that the improvement of a novel motor skill is accompanied by the formation of CCi spines, not TCi spines, during the initial 4-day training period.

Although the TCi spine formation rate itself was not significantly affected by the skill improvement (Fig. 2, C and F), the surrounding spine formation dynamics were different between the bilateral M1s (fig. S6). The dendritic segments with new TCi spines in cM1 showed higher spine formation activities [total numbers per length of spines formed during the 4-day training including transiently formed spines; note that new TCi spines were not counted as new spines in this analysis; see Supplementary Text] than those in iM1 and than the segments without new TCi spines in cM1 as well (fig. S6, A to C). New TCi spines frequently appeared in dendritic segments with new CCi spines formed during the 4-day training in cM1 (fig. S6, E and F). These analyses indicate that new TCi spines are preferentially formed at the dendritic segments with frequent new CCi spine formation during acquisition of the new motor skill. In addition, the high spine formation activity preceded the generation of new TCi spines that were sustained until day 4 (fig. S6, G to I), suggesting that active spine formation in the dendritic segment affects TCi spine formation there.

### Blocking of CC inputs to M1 impairs motor skill improvement

**Fig. 3.**
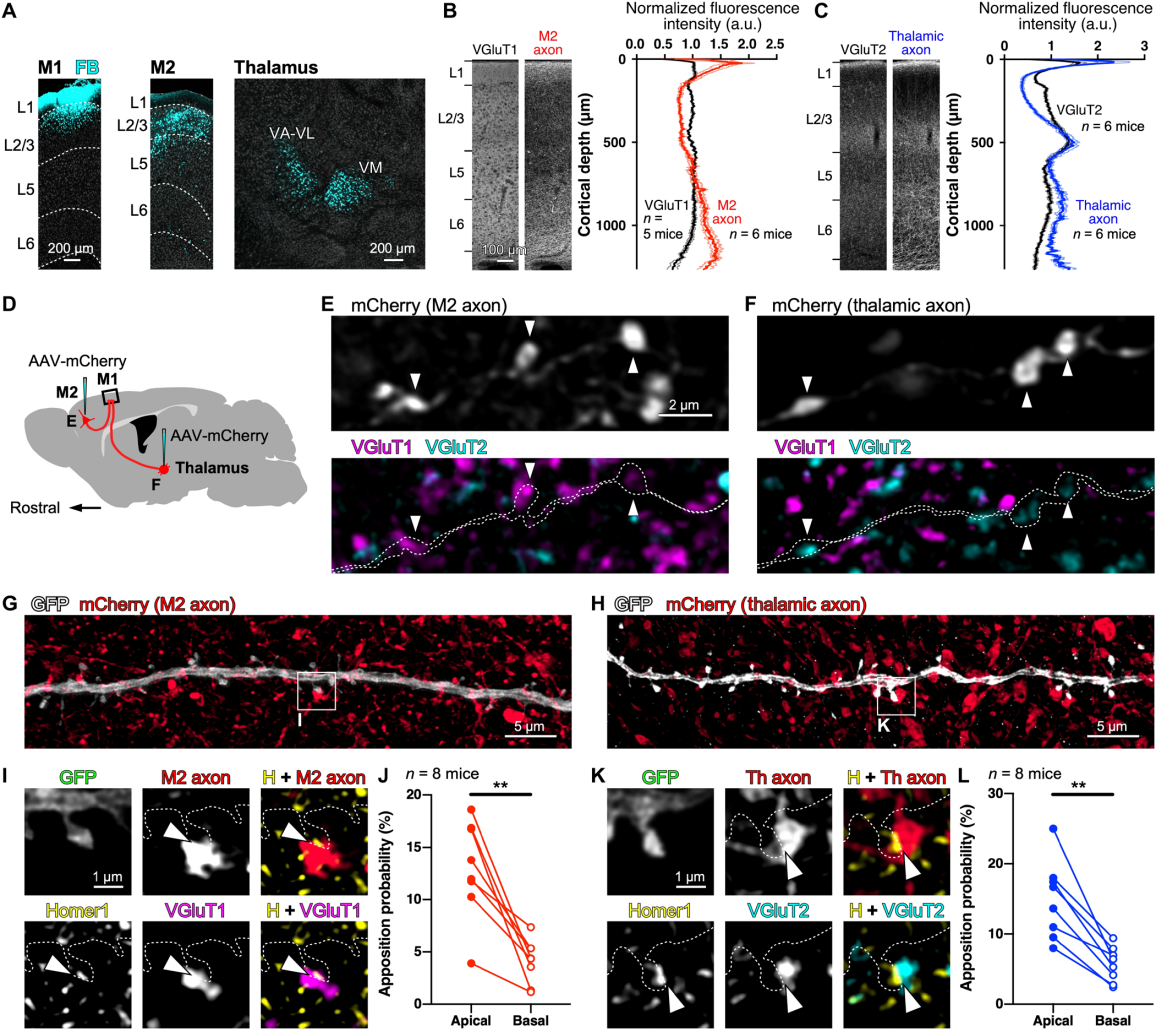
M2 and thalamic axons substantially form putative excitatory synapses on spines in the apical tufts. (**A**) Retrograde labeling of neurons that project to layer 1 of M1. Fast Blue (FB) was infused into layer 1 of M1 (left), retrogradely labeling cell bodies in M2 (middle) and the thalamus (right). (**B** and **C**) Fluorescence signals (means ± SEM) of VGluT-immunoreactive puncta, M2 axons, or thalamic axons in M1. Intensities of fluorescence signals within 1 mm from the cortical surface are normalized to 1 a.u. L, layer. (**D**) Schematic diagram of AAV-mCherry injection into M2 or the motor thalamus for anterograde labeling. (**E** and **F**) Triple-fluorescence labeling of mCherry [gray; (E), M2 axon; (F), thalamic axon], VGluT1 (magenta), and VGluT2 (cyan). The axon fibers labeled with mCherry are outlined by dotted lines. Arrowheads indicate axonal varicosities (boutons). (**G** and **H**) Fluorescence images of GFP-labeled dendrites (gray) of Thy1-eGFP-M line mice, M2 (G) and thalamic (H) axon fibers (red). (**I**) Confocal images of a GFP-labeled spine, an mCherry-labeled M2 bouton (red), a Homer1-immunoreactive signal in the spine head (yellow), and a VGluT1 immunofluorescence punctum (magenta). Arrowheads indicate a putative synaptic input site. (**J**) Apposition (putative synapse) probabilities of M2 axons in spines on the apical tufts and the basal dendrites of individual pyramidal cells (connected with lines). (**K**) Confocal images of a GFP-labeled spine, an mCherry-labeled thalamic bouton (red), a Homer1-immunoreactive signal in the spine head (yellow), and a VGluT2 immunofluorescence punctum (cyan). Arrowheads indicate a putative synaptic input site. (**L**) Apposition probabilities of spines contacted by thalamic boutons with Homer1-immunoreactive puncta in the apical tufts and the basal dendrites. **P < 0.01.

The postsynaptic spine plasticity can be affected by the presynaptic activity (*40*–*42*). Since the formation rate of the apical tuft spines was correlated with the skill improvement (Fig. 1E), the neurons that innervate the apical tufts of layer 5 pyramidal cells in M1 may be involved in motor learning here. Layer 1 of M1 is known to receive abundant excitatory inputs from the higher-order motor area, such as the secondary motor cortex (M2), and the motor-related thalamic nuclei (*30*–*34*). We identified numerous cell bodies projecting to the superficial layer of M1 in both M2 and the thalamus by local application of a retrograde tracer, Fast Blue (Fig. 3A and fig. S7). Although a subset of neurons in the somatosensory cortex were retrogradely labeled, CPn cells in M1 are reported to receive only weak input from the somatosensory cortex (*29*, *43*). Neurons in the ventral medial thalamic nucleus (VM), the ventral anterior–ventral lateral nuclear complex (VA-VL), and the anteromedial and the posterior nuclei were also labeled with the tracer. Anterograde labeling of the presynaptic M2 or thalamic neurons by injecting an adeno-associated virus (AAV) vector encoding mCherry into M2 or the thalamus visualized dense axonal projections to layer 1 in M1 from both M2 and the thalamus (Fig. 3, B and C). Compared with immunofluorescence signals for VGluTs, M2 and the motor thalamic nuclei preferentially targeted layer 1 of M1, indicating that M2 and motor thalamic neurons are the major input sources in layer 1. As reported previously (*44*–*46*), VGluT1 and VGluT2 immunoreactivities were selectively localized in anterogradely labeled CC and TC axonal varicosities in layer 1 [VGluT1/M2 bouton = 98.8 ± 0.4% (1133 of 1148) and VGluT2/M2 bouton = 2.2 ± 0.4% (27 of 1148), mean ± SEM; *n* = 6 mice; VGluT1/thalamic bouton = 1.1 ± 0.4% (12 of 1043) and VGluT2/thalamic bouton = 98.5 ± 0.7% (1027 of 1043), *n* = 6 mice; Fig. 3, D to F]. Conversely, anterograde labeling by virus vector injection can visualize only a subset of cortical and thalamic axons [M2 bouton/VGluT1 = 30.0 ± 0.8% (663 of 2248), *n* = 6 mice; thalamic bouton/VGluT2 = 46.7 ± 3.6% (464 of 956), *n* = 6 mice]. Although the distributions were slightly different between CC and TC axons, we could not find any statistical difference of cortical depth–dependent localization between CCi and TCi spines within 40 μm from the cortical surface (fig. S8).

Simultaneous imaging of postsynaptic dendritic spines (GFP), postsynaptic structure marker (Homer1), and presynaptic axons (mCherry) revealed that the axon fibers from M2 or the thalamus made a considerable number of synaptic appositions on dendrites of pyramidal cells (Fig. 3, G and H). The apposition probabilities on the spines of the apical tufts markedly differed from those on the spines of the basal dendrites (Fig. 3, I to L). The spines on the apical tufts were contacted about threefold more frequently than the spines on the basal dendrites by both M2 and thalamic axons [M2 axon: 12.98 ± 1.66% (318 of 2488 spines were innervated by M2 axons) of spines on the apical tuft and 4.10 ± 0.74% (238 of 6022) on the basal dendrites, mean ± SEM; *W* = −36, *P* = 0.0078, two-tailed Wilcoxon matched-pairs signed-rank test, *n* = 8 cells; thalamic axon: 14.89 ± 1.96% (371 of 2758 spines were innervated by thalamic axons) on the apical tuft and 5.63 ± 0.88% (371 of 7081) on the basal dendrites, *W* = −36, *P* = 0.0078, *n* = 8 cells; Fig. 3, J and L]. Thus, both of these CC and TC axons substantially targeted the spines on the apical tufts of layer 5 pyramidal cells.

**Fig. 4.**
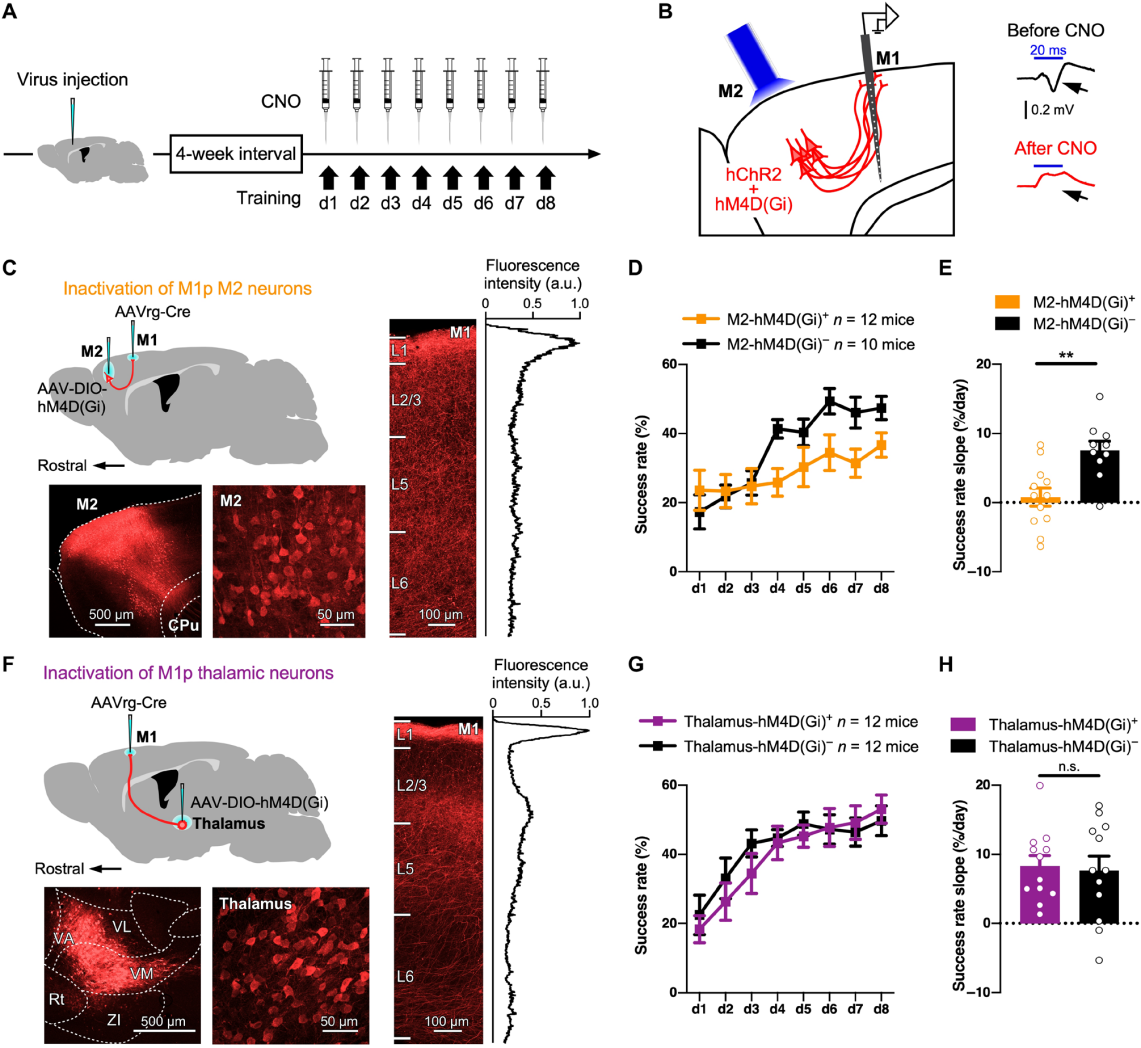
Chemogenetic silencing of M1p M2 neurons impairs motor learning. (**A**) Timeline of training with chemogenetic inactivation. (**B**) Left: Recording of M1 response evoked by M2 neuron photostimulation. A mixture of AAV-DIO-hChR2 and AAV-DIO-hM4D(Gi) was injected into M2, combined with injection of AAVrg-Cre into M1. Four weeks after the virus injection, the response in M1 neurons to optogenetic stimulation of M2 with blue light was measured with a 16-channel probe inserted into M1. Right: Evoked potentials at 100 μm below the pial surface by optogenetic stimulation of M2 neurons before (black trace) and 30 min after systemic CNO injection (red trace). Each trace represents the average over 243 measurements. The blue line indicates the duration of blue light (20 ms). Negative field potential reflects excitation. Also see figs. S9 and S10. (**C**) Virus injection for hM4D(Gi) expression in M1p M2 neurons. The peak fluorescence intensity of M2 axons in M1 is normalized to 1 a.u. CPu, caudate-putamen. (**D**) Success rate change during learning with or without chemogenetic silencing of M1p M2 neurons by systemic application of CNO (means ± SEM). (**E**) Success rate slopes during the initial 4 days of training with or without chemogenetic inactivation of M1p M2 neurons (means ± SEM). Each open circle represents a success rate slope of a mouse. (**F**) Virus injection for hM4D(Gi) expression in M1p thalamic neurons. The peak fluorescence intensity of thalamic axons in M1 is normalized to 1 a.u. Rt, reticular thalamic nucleus; ZI, zona incerta. (**G**) Success rate change during learning with or without chemogenetic silencing of M1p thalamic neurons (means ± SEM). Th, thalamus. (**H**) Success rate slopes during the initial 4 days of training with or without chemogenetic silencing of M1p thalamic neurons (means ± SEM). **P < 0.01. n.s., not significant.

The activities of these CC and TC neurons that innervate layer 1 of M1 may affect the motor skill acquisition. We therefore monitored changes in the success rate during 8 days of forelimb training with chemogenetic silencing of M1-projecting (M1p) M2 or thalamic neurons (Fig. 4 and figs. S9 and S10). We injected a retrograde (rg) AAV vector expressing Cre recombinase into M1 and another AAV vector that Cre-dependently expresses the chemogenetic receptor hM4D(Gi) in M2 or thalamic neurons (Fig. 4, B, C, and F). This virus injection labeled 21.3 ± 2.2% (825 of 3957 cells), 15.5 ± 1.7% (630 of 4343 cells), and 10.4 ± 1.7% (450 of 4606 cells) of layer 2/3, 5, and 6 neurons in M2, respectively (means ± SEM; *n* = 11 mice; fig. S9B), and 35.6 ± 2.6% (827 of 2377 cells) of thalamic neurons at the injection sites (*n* = 12 mice; fig. S10B). Both M1p M2 and thalamic neurons abundantly sent axons to layer 1 of M1 (Fig. 4, C and F). In control experiments, an AAV vector that Cre-dependently expresses channelrhodopsin 2 was also injected into M2 or the thalamus. Depolarization evoked by optogenetic M2 activation was specifically observed in the superficial layer of M1 (fig. S9D), consistent with layer 1–targeting projection of M2 → M1 axons (Fig. 4C). Either M1p M2 or thalamic neurons were silenced through systemic clozapine *N*-oxide (CNO) administration before every training step for 8 days [note that CNO was injected into both hM4D(Gi)^+^ and hM4D(Gi)^−^ mice; Fig. 4A]. We confirmed that CNO administration suppressed the evoked potential in M1 by optogenetic stimulation of the presynaptic neurons (Fig. 4B and figs. S9, C to E, and S10, C and D).

Compared with control animals (*n* = 10 mice) in which M1p M2 neurons expressed mCherry without hM4D(Gi), silencing of M1p M2 neurons (*n* = 12 mice) hindered the success rate increase (Fig. 4D) and resulted in slower learning as measured by the decrease in the success rate slope during the initial 4-day training (*U* = 16, *P* = 0.0025, two-tailed Mann-Whitney test; Fig. 4E). By contrast, silencing of M1p thalamic neurons did not significantly affect the motor learning (*n* = 12 mice; Fig. 4G), and the success rate slope was comparable to the control (*n* = 12 mice; *U* = 70.5, *P* = 0.9436; Fig. 4H). We thought that the effect of CNO was on motor skill acquisition (rather than, for example, motivation), because the reaching attempt frequency over all days was not affected by the suppression of M2 or the motor thalamus using direct chemogenetic inactivation of these regions (fig. S11).

**Fig. 5.**
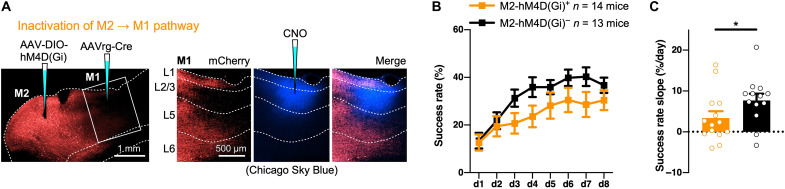
Selective blockade of the M2 → M1 pathway impairs skill improvement. (**A**) Left: Virus injection for hM4D(Gi) expression in M1p M2 neurons. Right: CNO was locally injected into the superficial layer of M1 before individual training sessions. The CNO-injected site was marked with Chicago Sky Blue 6B (pseudocolored blue) injected after the final training session. (**B**) Success rate change during learning with M2 → M1 pathway–selective blockade by local application of CNO (means ± SEM). (**C**) Success rate slopes during the initial 4 days of training with or without M2 → M1 pathway–selective blockade (means ± SEM). *P < 0.05.

Systemic CNO application could affect multiple cortical and subcortical projections from M2. To evaluate the direct effect of M2 → M1 innervation on the task learning, we monitored change in the success rate with pathway-selective inactivation of M2 → M1 projection by local application of CNO to the superficial layer of M1 (Fig. 5A). The local application of CNO before every training session hindered the skill improvement (Fig. 5B), which is implied by the significant decrease in the success rate slope [hM4D(Gi)^+^, *n* = 14 mice; hM4D(Gi)^−^, *n* = 13 mice; *U* = 49.5, *P* = 0.0436, two-tailed Mann-Whitney test; Fig. 5C]. These results indicate that CC inputs to M1 are of great importance for motor skill acquisition, consistent with the selective formation of CCi spines, not TCi spines, in motor skill acquisition (Fig. 2).

### Transient formation of new CCi spines versus persistent formation of new TCi spines

**Fig. 6.**
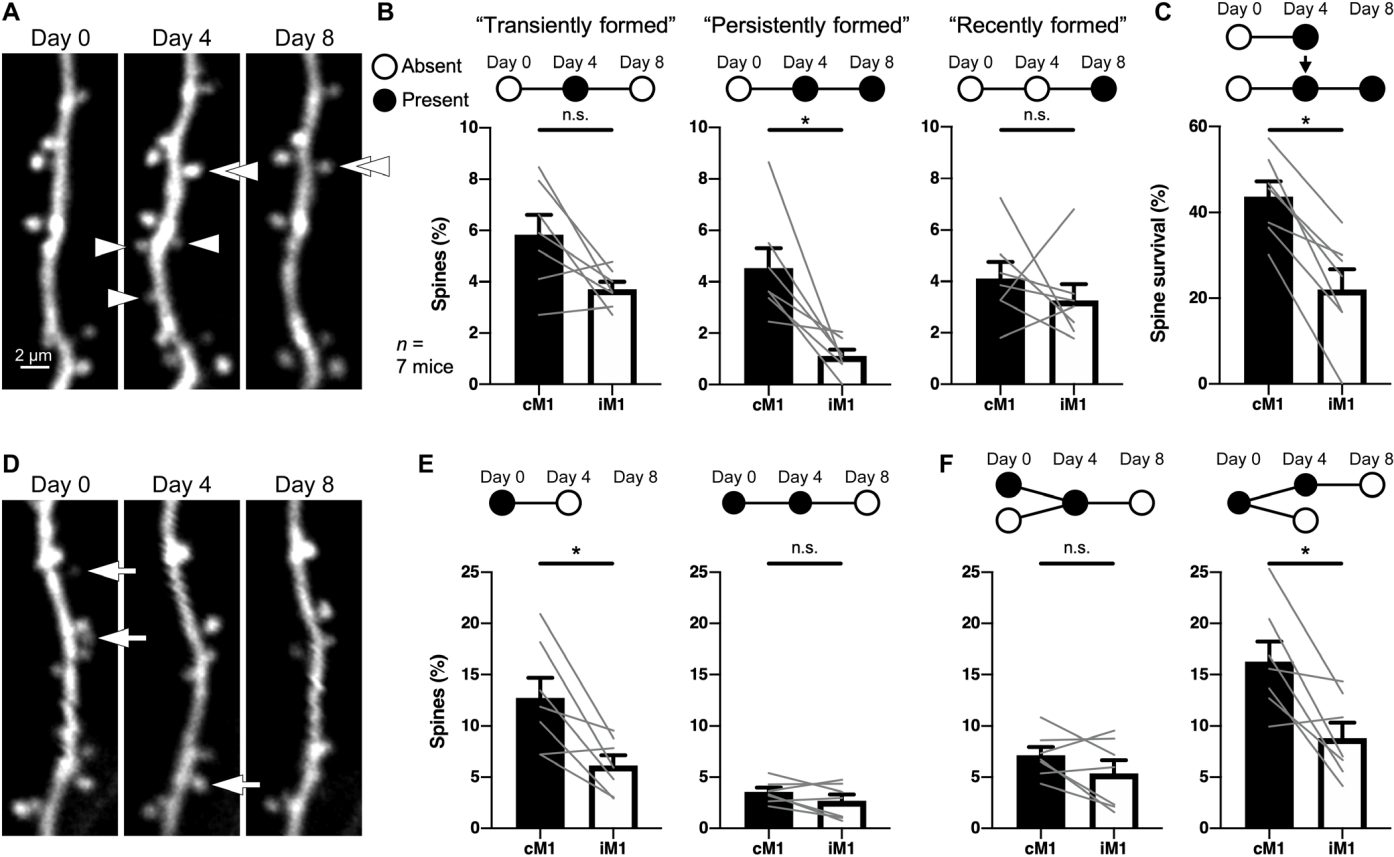
Spine dynamics during the 8-day task learning in the apical tufts of M1. (**A**) Two-photon images of spine formation in vivo at days 0, 4 and 8. Arrowheads indicate transiently formed spines, while double arrowheads indicate a persistently formed spine. (**B**) Formation rates of transiently, persistently, and recently formed spines (means ± SEM). Gray lines show formation rates of individual mice. (**C**) Survival ratio of new spines that formed by day 4 and retained until day 8 (means ± SEM). Gray lines show spine survival ratios of individual mice. (**D**) Two-photon images of spine elimination in vivo at days 0, 4, and 8. Arrows indicate eliminated spines. (**E**) Spines eliminated during the initial 4 days (left) and the following 4 days (right) (means ± SEM). Gray lines show data of individual mice. (**F**) Spines that were observable at day 4 and eliminated by day 8 (left) and spines that were observable at day 0 and eliminated by day 8 (right) (means ± SEM). *P < 0.05.

In addition to spine formation, maintenance of learning-associated new spines is assumed to be essential for long-term retention of motor memory (*1*, *2*). The long-term contribution of newly formed spines to the acquired motor memory should depend on their survival. We therefore extended the experimental period to 8 days and performed in vivo observation at days 0, 4, and 8 (Fig. 6). Mice with more than a 10% increase in success rate during the initial 4 days (success rate slope > 3.3) were included in the following analysis (*n* = 1276 spines in cM1 and 1188 spines in iM1, from seven mice). We classified the newly formed spines into three groups: new spines found at day 4 but eliminated by day 8 (transiently formed spines), new spines found at both days 4 and 8 (persistently formed spines), and new spines unobservable at day 4 but found at day 8 (recently formed spines). We found that persistently formed spines were significantly more frequently observed in cM1 than in iM1, and transiently formed spines also tended to be frequently observed in cM1, although not statistically significant (*W* = −28, *P* = 0.0156, two-tailed Wilcoxon matched-pairs signed-rank test; transiently formed, *W* = −22, *P* = 0.0781; recently formed, *W* = −10, *P* = 0.4688; Fig. 6, A and B). The spine survival ratio (the number of persistently formed spines divided by the total number of transiently and persistently formed spines) was also higher in cM1 (*W* = −28, *P* = 0.0156, two-tailed Wilcoxon matched-pairs signed-rank test) (Fig. 6C). These results indicate that the skill practice maintained the newly formed spines in cM1.

Intriguingly, elimination of the spines that were found at day 0 predominantly occurred during the first 4 days of the training (Fig. 6, D to F). The spine elimination rate during the initial 4 days was significantly higher in cM1 than in iM1 (Fig. 6E), whereas the elimination rate of the spines that survived for 4 days during the subsequent 4 days was low, and there was no significant difference between cM1 and iM1 (Fig. 6E). This result indicates that a large proportion of spines are stable, and spines maintained for 4 days are likely to persist thereafter.

**Fig. 7.**
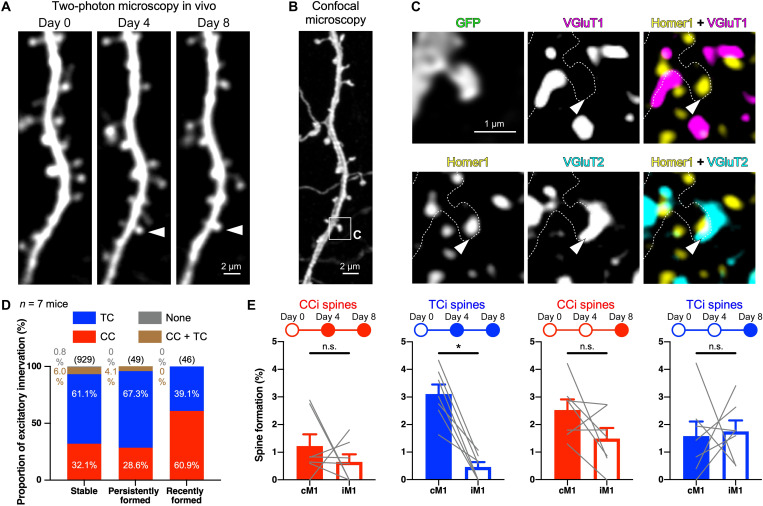
Prolonged learning maintains newly formed TCi spines but not CCi spines. (**A**) Two-photon images of spine dynamics in vivo at days 0, 4, and 8. Arrowheads indicate a persistently formed spine. (**B**) Confocal image of the corresponding dendritic segment to the two-photon images in (A). (**C**) High-magnification images of the square in (B). Arrowheads indicate a TC input site. (**D**) Proportion of excitatory innervations to stable, persistently formed, and recently formed spines. Values in parentheses represent the number of spines analyzed. (**E**) Spine formation rates of persistently formed and recently formed spines with CC or TC inputs (means ± SEM). Gray lines show the data of individual mice. *P < 0.05.

Post hoc immunohistochemistry (Fig. 7, A to C) revealed that the proportion of CC and TC inputs in the persistently formed spines (which were formed by day 4 and maintained until day 8) was comparable to that in stable spines (which were observable at all days 0, 4, and 8) (Fig. 7D) but different from that in the spines formed during the initial 4 days (newly formed spines in the 4-day training; Fig. 2D). The formation rate of persistently formed TCi spines was significantly higher in cM1 than in iM1 (*W* = −28, *P* = 0.0156, two-tailed Wilcoxon matched-pairs signed-rank test); in contrast, unexpectedly, there was no significant difference in the formation rate of persistently formed CCi spines between cM1 and iM1 (*W* = −12, *P* = 0.3750, two-tailed Wilcoxon matched-pairs signed-rank test) (Fig. 7E). Recently formed spines appeared with no significant difference between the bilateral M1s (CC, *W* = −20, *P* = 0.1094; TC, *W* = 4, *P* = 0.8125, two-tailed Wilcoxon matched-pairs signed-rank test), but 60.9% of them received inputs from CC axons (Fig. 7D) such as newly formed spines in the 4-day training (Fig. 2D). Although the presynaptic partners of the transiently formed spines could not be determined, taking the 4-day and 8-day experiments together, we estimate that 82.4% of CCi spines that formed by day 4 were eliminated by day 8, while only 9.4% of TCi spines disappeared by day 8 (see Supplementary Text and table S1). More transient formation of new CCi spines and longer survival of new TCi spines suggest that new CCi spines related to the skill improvement do not contribute to the skill retention, and new TCi spines are sustained as a result of motor learning.

### Shrinkage and enlargement of new CCi and TCi spines

EM (figs. S12 and S13 and movie S2) (*47*) displayed a synaptic structure on both nascent (<4 days after formation, *n* = 6) and persistent (≥4 days, *n* = 9) new spines except filopodium-like protrusions, which were excluded from the present study. In addition, three-dimensional (3D) reconstruction of serial EM images showed that the head volume of the new spines was much smaller than that of the stable spines (*n* = 83 stable spines and 15 newly formed spines; *U* = 188, *P* < 0.0001, two-tailed Mann-Whitney test; fig. S13E), as well as the bouton volume of presynaptic axons on the new spines (*U* = 221, *P* < 0.0001, two-tailed Mann-Whitney test; fig. S13F), indicating the immaturity of the new synapses that formed during the 8-day practice. Since spine size and synaptic strength are correlated (*48*, *49*), this suggests that new spines may need to further enlarge their size for stabilization of the acquired skill.

**Fig. 8.**
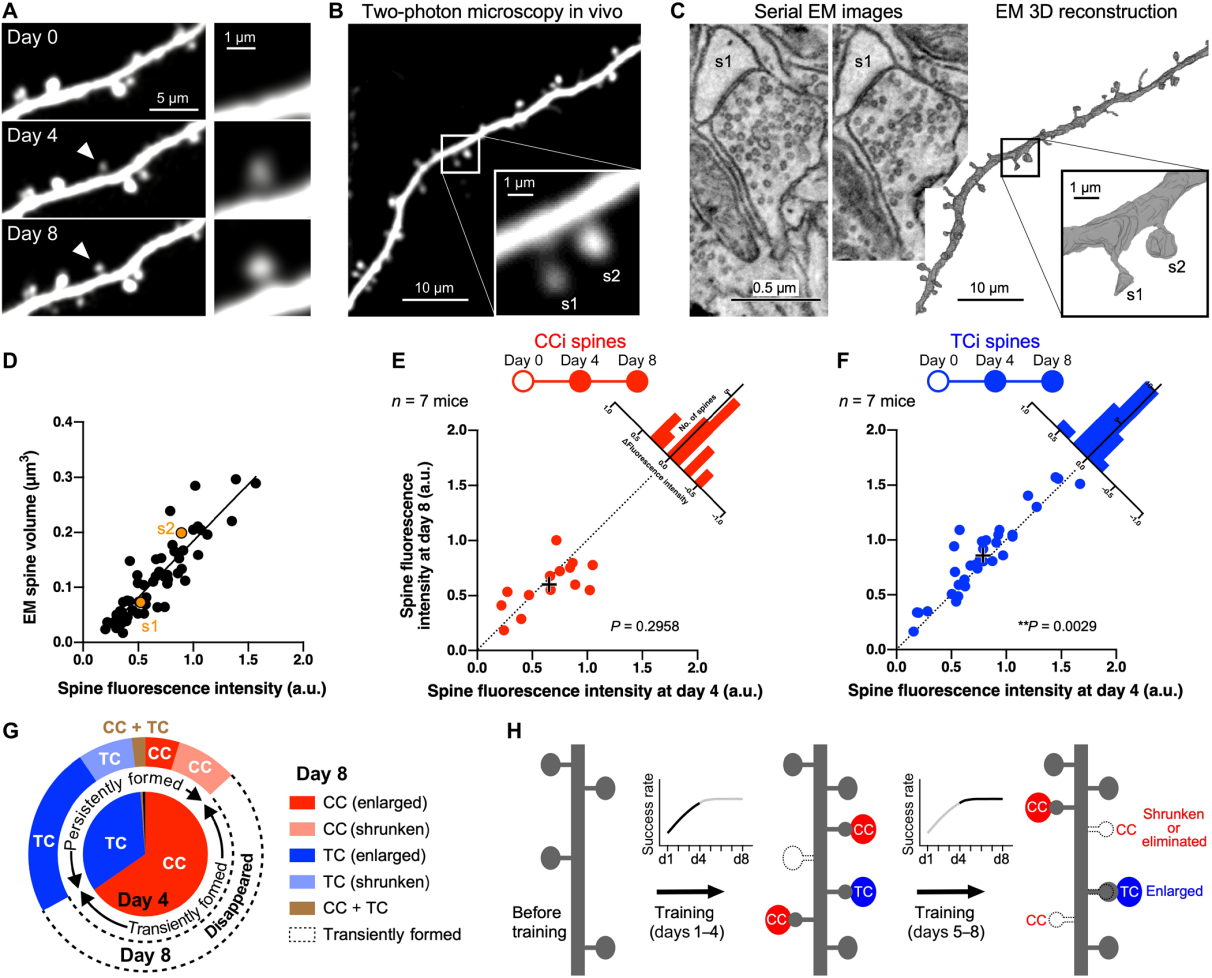
Elimination or shrinkage of new CCi spines versus sustained enlargement of new TCi spines from days 4 to 8. (**A**) Fluorescence fluctuation of a new spine. Arrowheads indicate a persistently formed new spine. (**B**) Dendritic segment observed in vivo under a two-photon microscope at day 8. (**C**) Serial EM images (left) and EM 3D reconstruction of the dendritic segment corresponding to the two-photon image in (B) (right). (**D**) Correlation of spine fluorescence intensities normalized to shaft intensities with spine head volumes measured in EM reconstructions. (**E** and **F**) Correlations of CCi (E) and TCi (F) spine fluorescence intensities at days 4 and 8 in the persistently formed spines. Histograms show the distributions of pairwise differences. (**G**) Pie chart showing the proportion of new spines that formed by day 4. The inner pie chart shows the proportion of inputs to new spines analyzed in the 4-day experiment (also see Fig. 2D). The outer pie chart represents the proportion of transiently and persistently formed spines in the 8-day training (also see Fig. 7D), and persistently formed spines are classified on the basis of the presynaptic origin and spine enlargement/shrinkage between days 4 and 8. (**H**) Schematic diagram of spine dynamics during motor learning.

Spine enlargement is associated with long-term potentiation of synapses in an activity-dependent manner (*49*–*53*). Fluorescence intensity of spines was reported to represent spine volume (*53*, *54*), and we observed fluctuations of fluorescence intensities in some new spines during learning (Fig. 8A). Since spine fluorescence intensity normalized to shaft intensity was linearly correlated with spine volume measured by EM 3D reconstruction (*n* = 57, *r* = 0.8895, *P* < 0.0001, Pearson correlation coefficient; Fig. 8, B to D, and fig. S14), we compared the spine fluorescence intensities of the persistently formed CCi and TCi spines between days 4 and 8. The fluorescence intensity of survivor CCi spines did not significantly differ (*W* = −35, *P* = 0.2958, two-tailed Wilcoxon matched-pairs signed-rank test), whereas that of TCi spines showed a slight but statistically significant increase from days 4 to 8 (*W* = 325, *P* = 0.0029, two-tailed Wilcoxon matched-pairs signed-rank test) (Fig. 8, E and F). The changes in fluorescence intensity in the spine heads (Δfluorescence intensity = *I*_d8_ − *I*_d4_; *I*, normalized spine fluorescence intensity) showed a significant difference between persistently formed CCi and TCi spines (*D* = 0.4610, *P* = 0.0306, Kolmogorov-Smirnov test; Fig. 8, E and F), indicating that new CCi spines were less likely enlarged from days 4 to 8 than new TCi spines. It could be consequently assumed that a majority of new CCi spines were formed only transiently or had shrunken their spine heads (Fig. 8G). On the other hand, most of the new TCi spines were maintained until day 8 with spine enlargement (Fig. 8G). These results imply that newly formed TC synapses are more persistent and strengthened than CC synapses after 8 days of practice, consistent with a previous report that showed an increase in amplitude of TC inputs after motor learning (*17*). These input-dependent spine dynamics (Fig. 8H) suggest unique contributions of CC and TC afferents in M1 to motor learning.

### Chemogenetic silencing of M1p M2 neurons reduces spine formation in M1

**Fig. 9.**
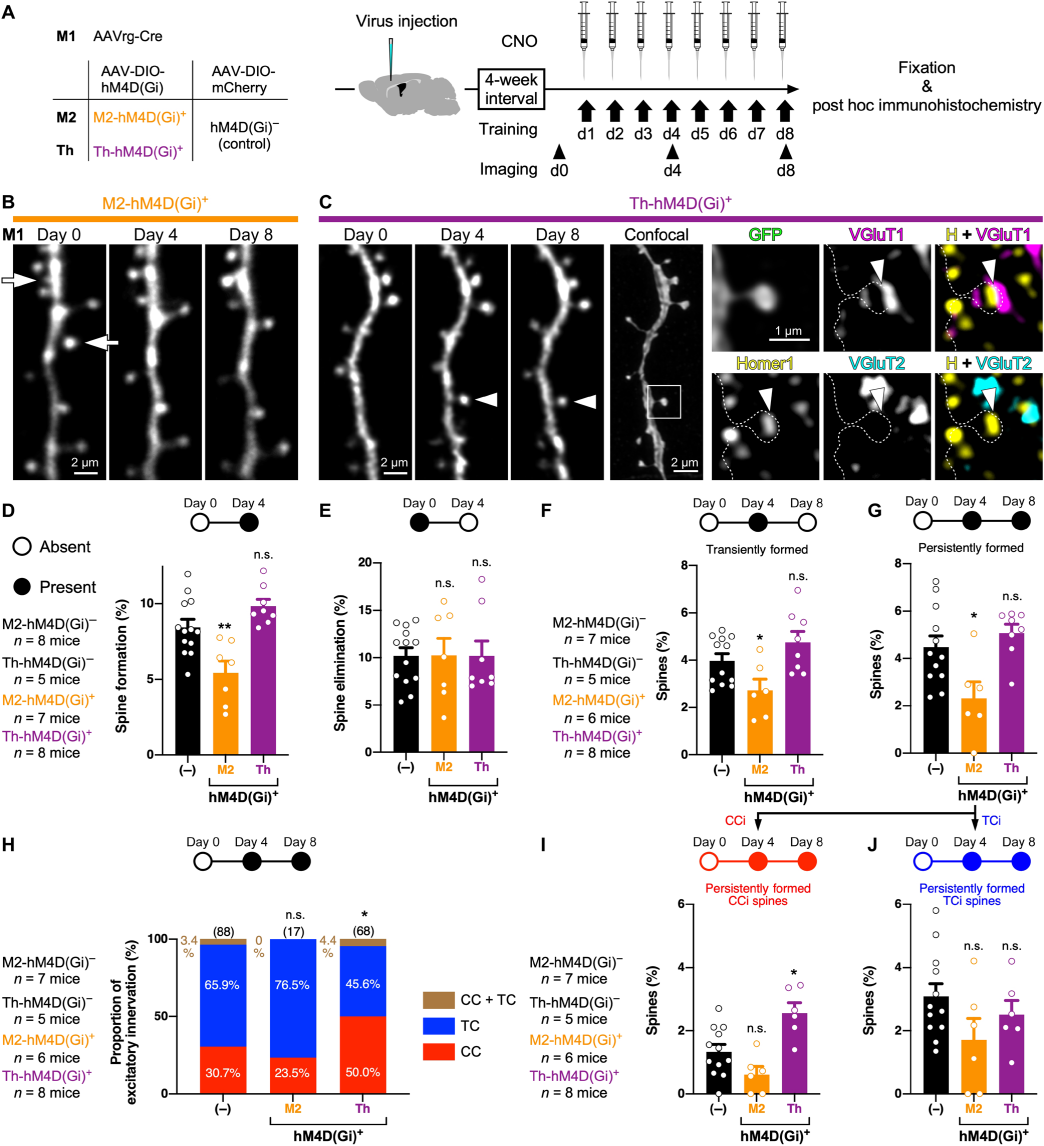
Chemogenetic blockade of CC and TC inputs to M1 differently perturbs spine dynamics during skill training. (**A**) Timeline of task training and spine dynamics imaging under chemogenetic silencing. (**B**) Two-photon images of spine dynamics during motor learning under chemogenetic silencing of M1p M2 neurons. Arrows indicate eliminated spines. (**C**) Images of two-photon microscopy under chemogenetic silencing of M1p thalamic neurons followed by post hoc immunohistochemistry. Arrowheads in two-photon images indicate a persistently formed spine. Arrowheads in confocal images indicate a putative synaptic contact site. (**D** and **E**) Spine formation (D) and elimination (E) rates between days 0 and 4 with or without chemogenetic silencing (means ± SEM). Spine formation and elimination rates in control animals, the M2-hM4D(Gi)^+^ group, and the Th-hM4D(Gi)^+^ group are shown in black, orange, and purple, respectively. Open circles show data of individual animals. (**F** and **G**) Rates of transiently (F) and persistently formed (G) spines with or without chemogenetic silencing (means ± SEM). (**H**) Proportion of excitatory innervations to persistently formed spines in the hM4D(Gi)^−^ group, the M2-hM4D(Gi)^+^ group, and the Th-hM4D(Gi)^+^ group. Values in parentheses represent the number of spines analyzed. (**I** and **J**) Rates of persistently formed CCi (I) and TCi (J) spines with or without chemogenetic silencing (means ± SEM). *P < 0.05 and **P < 0.01.

We subsequently examined the effect of presynaptic activity on the spine formation in M1 during motor learning. As described above (Fig. 4, C and F), we injected AAVrg-Cre into M1 and AAV-DIO-hM4D(Gi) into M2 or the thalamus of Thy1-eGFP-M mice, and CNO was applied intraperitoneally before every training session for selective silencing of M1p M2 or thalamic neurons (Fig. 9A). In control mice, AAV-DIO-mCherry was injected into M2 or the thalamus instead of AAV-DIO-hM4D(Gi) [the hM4D(Gi)^−^ group]. Since spine dynamics were comparable between the M2-hM4D(Gi)^−^ (*n* = 8 mice) and Th-hM4D(Gi)^−^ (*n* = 5 mice) groups, the control data of both hM4D(Gi)^−^ groups were combined. In vivo two-photon imaging of the apical tufts in cM1 at days 0 and 4 of the task learning showed that the correlation between the success rate increase and spine dynamics (both formation and elimination) in the hM4D(Gi)^−^ group (*n* = 13 mice, 2405 spines in total) did not significantly differ from that in animals without CNO application (Fig. 1, E and G), indicating that systemic CNO application itself did not directly influence spine dynamics in M1 (fig. S15). On the other hand, the M2-hM4D(Gi)^+^ group (*n* = 7 mice, 926 spines in total; Fig. 9B) had a lower spine formation rate than the hM4D(Gi)^−^ group (*U* = 12, *P* = 0.0063, two-tailed Mann-Whitney test; Fig. 9D), whereas spine formation was not impaired in the Th-hM4D(Gi)^+^ group (*n* = 8 mice, 1829 spines in total); rather, the spine formation rate in the Th-hM4D(Gi)^+^ group tended to be larger than that in the control, although not statistically significant (*U* = 25, *P* = 0.0535; Fig. 9D). Unlike formation, the spine elimination rate in the M2-hM4D(Gi)^+^ group was comparable to that in the M2-hM4D(Gi)^−^ group (*U* = 42, *P* = 0.8168), similar to that in the Th-hM4D(Gi)^+^ group (*U* = 47, *P* = 0.7501; Fig. 9E). Observation of spine dynamics at days 0, 4, and 8 revealed that silencing M1p M2 neurons reduced both transiently (*U* = 12, *P* = 0.0229; Fig. 9F) and persistently formed spines (*U* = 12, *P* = 0.0245; Fig. 9G). The impairment of the spine formation as well as motor skill acquisition (Fig. 4, D and E) by silencing of inputs from M2 to M1 confirms the importance of CC inputs to new spines in M1 for learning during 4 days.

### Chemogenetic silencing of M1p thalamic neurons sustains new CCi spines in M1

Chemogenetic silencing of M1p thalamic neurons altered neither skill improvement (Fig. 4, G and H) nor the number of spines formed during learning (transiently formed spines, *U* = 29.5, *P* = 0.1625; Fig. 9F; persistently formed spines, *U* = 35, *P* = 0.3431; Fig. 9G). However, post hoc immunohistochemistry (Fig. 9C) revealed that the proportion of presynaptic cell types innervating persistently formed spines in the Th-hM4D(Gi)^+^ group differed from that in the control; about 30% of persistently formed spines received CC inputs in hM4D(Gi)^−^ mice, whereas half of those in the Th-hM4D(Gi)^+^ group were CCi spines [*P* = 0.0381, chi-square test; M2-hM4D(Gi)^+^ group, *P* = 0.5882; Fig. 9H]. Although chemogenetic silencing of M1p thalamic neurons did not significantly affect the persistent formation of TCi spines (*U* = 27, *P* = 0.4240; Fig. 9J), unexpectedly, the TC silencing resulted in generating more persistently formed CCi spines than the control (*U* = 11, *P* = 0.0171; Fig. 9I).

**Fig. 10.**
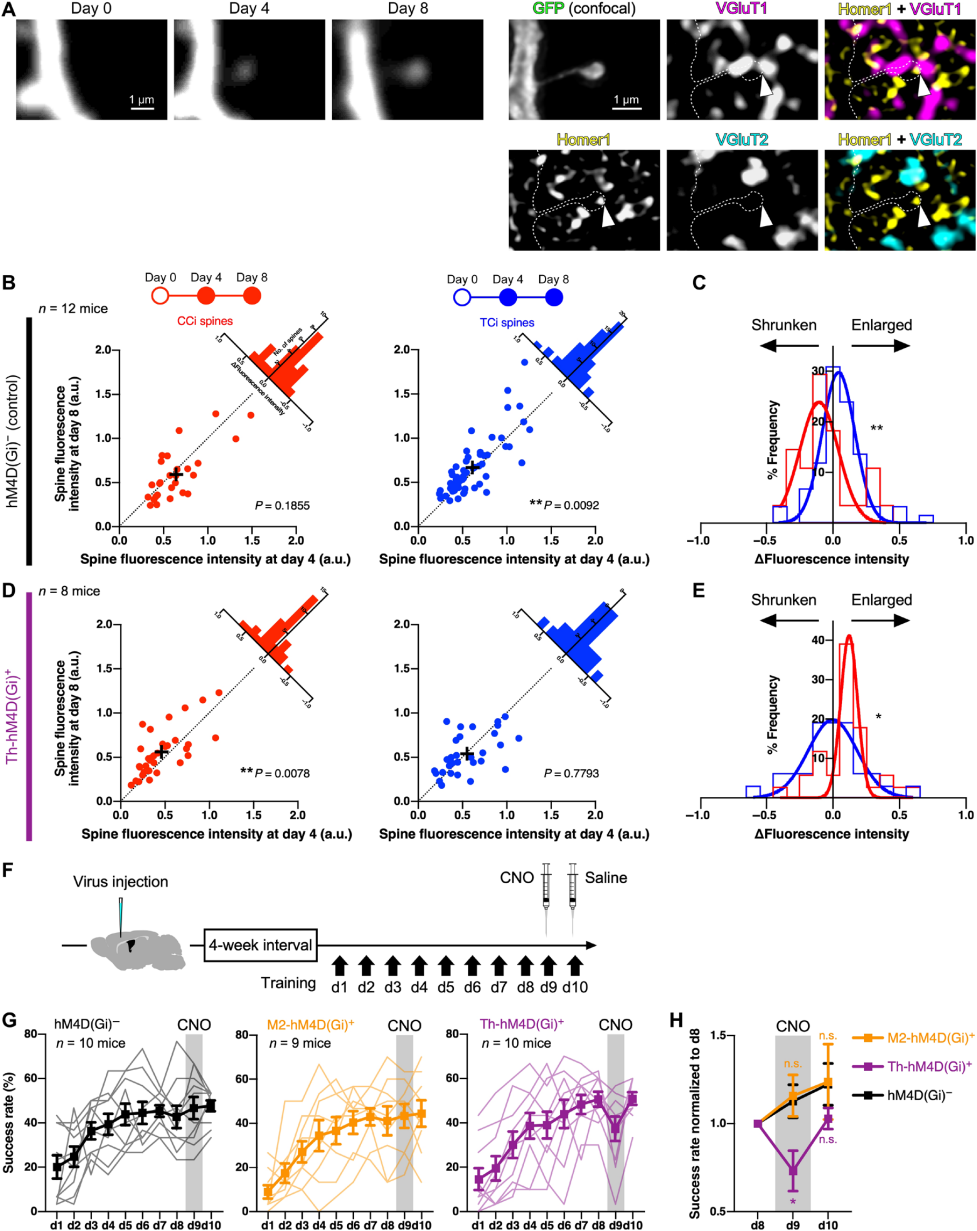
Chemogenetic blockade of TC inputs enlarges newly formed CCi spines. (**A**) Two-photon imaging of a newly formed CCi spine followed by post hoc immunohistochemistry. Spine fluorescence is enhanced from days 4 to 8. The spine forms a close contact with a VGluT1-immunoreactive punctum. (**B**) Normalized spine fluorescence intensities of persistently formed CCi and TCi spines between days 4 and 8 in hM4D(Gi)^−^ control mice. (**C**) Frequency histogram of Δfluorescence intensity (*I*_d8_ − *I*_d4_; *I*, normalized spine fluorescence intensity) of persistently formed CCi (red) and TCi (blue) spines. Fitted Gaussian distributions are also shown. (**D**) Normalized spine fluorescence intensities of persistently formed CCi and TCi spines between days 4 and 8 in Th-hM4D(Gi)^+^ control mice. (**E**) Frequency histogram of Δfluorescence intensity of persistently formed CCi (red) and TCi (blue) spines. Fitted Gaussian distributions are also shown. (**F**) Timeline of the 8-day training followed by chemogenetic silencing at day 9. Mice learned the task for 10 days, with CNO and saline injection at days 9 and 10, respectively. (**G**) Success rate changes in hM4D(Gi)^−^ control mice (left), M2-hM4D(Gi)^+^ mice (middle), and Th-hM4D(Gi)^+^ mice (right). Lines in light color show data of individual animals, while solid lines represent means ± SEM. (**H**) Success rates from days 8 to 10 normalized to those at day 8. *P < 0.05 and **P < 0.01.

Why was the generation of persistently formed CCi spines enhanced by the silencing of M1p thalamic neurons? We noticed that the fluorescence intensity of persistently formed CCi spines was also enhanced from days 4 to 8 in the Th-hM4D(Gi)^+^ group (Fig. 10A). In the hM4D(Gi)^−^ control mice, the fluorescence intensity of persistently formed TCi spines was significantly increased from days 4 to 8 (*W* = 667, *P* = 0.0092, two-tailed Wilcoxon matched-pairs signed-rank test), while that of persistently formed CCi spines was not changed between days 4 and 8 (*W* = −112, *P* = 0.1855) (Fig. 10B), similar to the result in mice without the virus injection (Fig. 8, E and F). ΔFluorescence intensities (*I*_d8_ − *I*_d4_; *I*, normalized spine fluorescence intensity) differed significantly between persistently formed CCi and TCi spines (*D* = 0.3857, *P* = 0.0083, Kolmogorov-Smirnov test; Fig. 10C). By contrast, in the Th-hM4D(Gi)^+^ group, the enlargement of TCi spines was prevented (*W* = −30, *P* = 0.7793), while CCi spines increased their fluorescence intensity (*W* = 293, *P* = 0.0078) (Fig. 10D). Consequently, the Δfluorescence intensity of persistently formed CCi spines was larger than that of TCi spines (*D* = 0.3744, *P* = 0.0226; Fig. 10E).

These results indicate that chemogenetic silencing of M1p thalamic neurons does not affect TCi spine formation but suppresses the enlargement and maturation of the persistently formed TCi spines; this presynaptic activity–dependent enlargement of new TCi spines may be necessary for pruning of newly formed CCi spines. It is also suggested that the suppression of TCi spine enlargement could be compensated by enhanced formation and enlargement of persistently formed CCi spines, allowing intact behavioral performance during learning under silencing of M1p thalamic neurons (Fig. 4, G and H).

From the input type–dependent spine formation/maturation patterns during skill improvement, we reasoned that the persistently formed TCi spines are related to memory consolidation. This possibility was tested by temporary chemogenetic suppression of CC or TC inputs in expert mice after skill improvement. We trained mice for 8 days without CNO administration and intraperitoneally injected CNO 30 min before the training session at day 9 (Fig. 10F). The following day (day 10), the same amount of saline was injected before the task. CNO administration did not alter the success rate either in the hM4D(Gi)^−^ group [*n* = 5 M2-hM4D(Gi)^−^ mice and 5 Th-hM4D(Gi)^−^ mice] or in the M2-hM4D(Gi)^+^ group (*n* = 9 mice) (Fig. 10G). On the other hand, CNO application at day 9 in the Th-hM4D(Gi)^+^ group decreased the success rate (*n* = 10 mice; Fig. 10G). The success rate at day 9, which normalized to that at day 8, was significantly lower in the Th-hM4D(Gi)^+^ group than in the control [*P* = 0.0162, Kruskal-Wallis test; M2-hM4D(Gi)^+^ group, *P* > 0.9999; Th-hM4D(Gi)^+^ group, *P* = 0.0380, Dunn’s multiple comparisons test] (Fig. 10H). The success rate could be recovered at day 10 with saline injection [*P* = 0.4964, Kruskal-Wallis test; M2-hM4D(Gi)^+^ group, *P* = 0.8197; Th-hM4D(Gi)^+^ group, *P* = 0.5060, Dunn’s multiple comparisons test]. This result indicates that expert mice no longer require top-down innervation from M2 to M1, whereas established TC innervation predominantly allows skillful movement acquired through learning. Thus, in M1, acquired motor memory may be stored at persistently formed TCi spines.

## DISCUSSION

In this study, we analyzed dendritic spine dynamics and identified their presynaptic neuronal partners during motor skill learning. We report a bifurcation of spine connectivity during learning. First, top-down CC neurons made profuse, small, and transient contacts with spines during the learning asymptote. Second, TC neurons formed fewer but larger and longer-lasting contacts as learning reached asymptote. This division of labor of spine regulation indicates that afferents from different presynaptic neurons may be specialized for distinct roles in motor learning. Spine plasticity is observed throughout the neocortex (*55*), and cortical layer 1 receives convergent inputs from both higher-order cortical areas and the subcortical thalamus. The transition in sensorimotor skill learning from top-down, goal-directed practice during the training period to an automatic and habitual regulation after learning is consistent with the dual presynaptic circuit supervision we report, which may represent a canonical neocortical process for other skillful learned behaviors.

Here, we summarize the characterization of presynaptic inputs to new spines on M1 neurons formed during motor learning. We found unique dynamics of new CCi and TCi spines, respectively (Fig. 8H). Spine formation correlated with skill improvement, and new spines received CC inputs more frequently than stable spines (Fig. 2D). This presynaptic source-dependent spine formation (Fig. 2, C to F) was supported by chemogenetic inactivation that showed a dependence on M1p M2 neurons, but not M1p thalamic neurons, for motor learning (Figs. 4 and 5). Spine formation in M1 was also hindered by chemogenetic silencing of M1p M2 neurons (Fig. 9, D, F, and G), suggesting that neural activity in presynaptic M2 cells innervating M1 influences spine formation. The predominant source of long-range CC input to layer 1 of M1 was presumably M2 neurons (Fig. 3A and fig. S7), and this top-down connection may be finely tuned during motor skill learning to entrain the excitability of the apical tuft dendrites of CPn-type pyramidal cells in M1, which output motor commands to subcortical regions. However, CC inputs were no longer necessary for expert mice, and TC inputs predominantly functioned for the acquired skill (Fig. 10G). Daily silencing of TC inputs during learning did not affect spine formation itself (Fig. 9, D, F, and G), but it impaired TCi spine enlargement (Fig. 10D). This suppression of TCi spine enlargement coincided with the enlargement of persistently formed CCi spines (Fig. 10, D and E), suggesting that maintenance of the motor memory can be compensated by the CC network (Fig. 4, G and H) and that pruning of newly formed CCi spines requires maturation of newly formed TCi spines.

New CCi spines were formed only transiently despite the importance of presynaptic M2 neurons for synaptogenesis in M1 as well as successful acquisition of the motor skill (Figs. 4, 5, and 9). It was recently shown that, in frontal-sensory pathways, top-down CC projections may represent prediction errors for behavioral adaptation through the functional remapping of responses to sensory stimuli in primary sensory areas (*56*, *57*). For example, during somatosensory reversal learning, top-down innervation from the lateral orbitofrontal cortex updates sensory representations in the primary somatosensory cortex (*56*): The lateral orbitofrontal cortex transiently shows large responses to unexpected outcomes after a rule switch. This function of top-down signals in sensory learning suggests that, in the case of motor learning as well, feedback signals from M2 neurons might provide essential training-dependent conditioning of relevant dendritic branches that updates internal programs for mnemonic transfer to stable synaptic connections from the thalamus to M1.

The formation of new CCi spines during the early learning period could play a role in reconfiguring neuronal circuitry. TCi spine formation in cM1 was less common than CCi spine formation and comparable to that in iM1. New TCi spines in cM1 that formed by day 4 were, however, mostly maintained until day 8, with enlarged spine head sizes (Fig. 8G). The selective TCi spine formation that we found in dendritic segments with high spine formation activity (fig. S6) is consistent with a study using the same seed reaching task reporting the clustering of synaptic inputs on the dendritic trees of layer 5 pyramidal cells that persisted for weeks to months (*3*). Clustered synaptic inputs can trigger local, *N*-methyl-d-aspartate–dependent dendritic spikes (*58*–*60*) in vitro (*61*, *62*) and in vivo (*63*) to drive action potential firing. Slice culture recordings observed glutamate receptor currents in new spines that formed even within a few hours (*64*). We propose that early local dendritic activity may facilitate the stabilization and selective strengthening of learning-induced new TCi spines (*65*). Active spine formation preceded the generation of TCi spines that persisted by day 4 (fig. S6, G to I). The reconfiguration of synaptic inputs during learning may depend on local dendritic activity triggered by the dynamic clustering of CC synaptic inputs, leading to the consolidation of TC synapses on dendrites and spines (*50*, *66*, *67*) in a branch-specific manner (*6*, *68*).

Post hoc characterization used in the present study could not satisfy the temporal resolution that allows the description of the precise dynamics of individual CCi and TCi spines. We could not identify the origin of inputs to new spines eliminated by day 4. Given that a large proportion of new spines found at day 4 received CC inputs, we can expect that many of the new spines eliminated by day 4 were also innervated by CC axons. However, there is also the possibility that new spines eliminated by day 4 were temporarily innervated by TC axons. We observed a relatively small number of new TCi spines at day 4, and they were mostly maintained. It is also possible that new TCi spines were sorted into “ephemeral” and “immortal” spines very early in learning, and only immortal TCi spines remained until day 4. In this case, the immortality might be affected by neighboring CCi spines (fig. S6, E and F). Further technical development may allow day-by-day identification of cell types innervating new spines. It would explain what causes the different properties of CCi and TCi spine dynamics and how nearby CCi and TCi spines mutually influence each other’s dynamics.

Chemogenetic silencing of M1p M2 neurons revealed a clear contribution of CC inputs to spine formation (Fig. 9D) and skill improvement (Figs. 4 and 5). However, temporary silencing at day 9 in expert mice (Fig. 10, F to H) suggests that feedback input from the higher-order cortex is no longer necessary for the performance of the expert mice (Fig. 10G, middle) and that the acquired motor memory was stored in the persistently formed TC circuit (Fig. 10G, right). Daily blockade of TC inputs seemingly did not directly affect either spine formation (Fig. 9D) or behavioral performance (Fig. 4, G and H). Post hoc immunohistochemistry showed almost intact formation of TCi spines in the Th-hM4D(Gi)^+^ mice (Fig. 9J). Suppression of presynaptic thalamic neuron activity, however, impaired spine size enlargement in TCi spines; alternatively, the spine enlargement was undertaken by CCi spines (Fig. 10D). In addition, the suppression of TCi spine enlargement might have caused the persistent formation of CCi spines (Fig. 9I). These results raise the possibility that maturation and stabilization of TCi spines formed through learning are necessary for pruning of learning-related new CCi spines. The intrinsic process of TCi spine enlargement with CCi spine pruning may allow transition from attentive to subliminal skill improvement in learning. Therefore, although task performance was seemingly unaffected in the Th-hM4D(Gi)^+^ mice (Fig. 4, G and H), it is possible that the mice needed to pay more attention to the task than the control mice.

In the present work, we focused on layer 5 CPn (or PT) cells in M1, which are essential for motor learning (*69*), but other cell subtypes in M1 are also related to movement. Layer 2/3 pyramidal cells and layer 5 intratelencephalic cells (IT cells; or crossed-corticostriatal cells) are also involved in motor control: In particular, the activity of layer 2/3 pyramidal cells is dynamically modulated during motor learning with enhanced spine dynamics in the apical tufts (*70*, *71*). Layer 2/3 cells also represent the success/failure outcome of trials that is developed through learning, and their activity that represents the outcome information of the previous action may partially influence the activity of layer 5 CPn cells in the next trial (*72*). In the prelimbic cortex, IT cells contribute to goal-directed learning through the corticostriatal pathway (*73*), suggesting that layer 5 IT cells in M1 are also involved in motor learning by interaction with the basal ganglia. Since layer 2/3 pyramidal cells and layer 5 IT cells can participate in motor learning, further studies will be needed to describe the entire remodeling of the afferent inputs to these cell types and to CPn cells.

In summary, our findings open a new perspective on the role of presynaptic input diversity to dendritic spines in learning new skills. Given that higher-order motor areas are associated with context-dependent motor commands, goal-directed behaviors, and motor planning (*12*, *19*, *20*, *74*), cognitively demanding processes during the early phase of learning involve top-down CC inputs to cortical layer 1 (*75*). In contrast, the motor-related thalamic nuclei are hubs for signal transmission from the basal ganglia and the cerebellum to the cortex (*21*, *22*), and TC afferents enable motor preparation and execution (*76*, *77*). The successful completion of motor learning depends on coordinated activity between cortical neuronal ensembles and subcortical neurons in the cerebellum and the dorsolateral striatum (*14*, *15*). Therefore, as TC afferents develop movement-locked dynamics (*16*), the acquired motor skill may be stored long term at TC innervation sites. The spine division of labor that we report, transient CCi versus persistent TCi spine formation, suggests that input type–dependent synaptic reconfiguration occurs as motor learning proceeds, where CC inputs to layer 1 specify TC inputs for long-term motor memory. Further studies will deepen and expand the role of presynaptic input diversity to spines during behavior.

## MATERIALS AND METHODS

### Experimental design

The single-seed reaching task (*1*) was adapted for in vivo imaging of spine dynamics during motor learning (Fig. 1A). After the individual training sessions, the apical tufts of layer 5 pyramidal cells were observed by two-photon microscopy (Fig. 1C). Immediately after the imaging at day 4 (Fig. 2) or day 8 (Fig. 6), mice were fixed followed by post hoc immunohistochemistry for presynaptic (VGluT1 for CC axons and VGluT2 for TC axons) and postsynaptic (Homer1) structure markers as well as GFP-labeled dendrites of Thy1-eGFP-M mice (figs. S2 and S3). The presynaptic partners of individual stable and new spines were identified manually, and the proportion of spines innervated by CC or TC axons was quantified. Combined with virus injection for hM4D(Gi) expression, the success rate of the motor task and the spine dynamics were monitored with chemogenetic blockade of CC or TC inputs to M1 (Figs. 4, 5, 9, and 10 and figs. S9 and S10). CLEM (fig. S12) confirmed the synaptic structure on the contact sites of spines and presynaptic markers (fig. S4) and the correlation between fluorescence intensity with two-photon microscopy and spine head size (Fig. 8).

### Animals

Four- to 8-week-old Thy1-eGFP-M mice on a C57BL/6J background (*78*) (68 mice; the Jackson Laboratory, Bar Harbor, ME) and C57BL/6J mice (124 mice; the Jackson Laboratory) (both males and females) were used. All animal care and use were in accordance with the guideline of the Institutional Animal Care and Use Committee of the National Institutes for Natural Sciences. Mice were housed individually on a 12-hour light/12-hour dark cycle. All efforts were made to minimize animal suffering and the number of animals used.

### Motor learning of the single-seed reaching task

The single-seed reaching task was applied according to previous reports (*1*, *3*). Mice were mildly food-restricted, with less than 10% body-weight loss before starting training. Mice were able to move freely in an acrylic transparent chamber (130 mm by 130 mm wide, 240 mm high). The training consisted of two phases: a shaping phase to familiarize the animals with the task and determine their preferred forelimb for reaching and a training phase to improve their skill. During the shaping phase (1 to 5 days), a small pile of millet seeds was placed in front of a 5-mm-wide slit, wide enough to reach one forelimb through. The preferred forelimb of each mouse was determined if more than 70% of 20 reaches within 30 min were attempted with one particular forelimb. Training started from the day after shaping completed. During the training phase, single seeds were placed one by one in front of a 5-mm-wide slit located at a distance of 20 mm from the wall on the side of the preferred forelimb, and each training session finished when mice attempted to reach seeds 30 times or spent 30 min. The success rate was the number of success in grasping seeds divided by total attempts. Training sessions were recorded by single-lens digital cameras (DSC-QX10 or ILCE-QX1, Sony, Tokyo, Japan) with a camera control application (PlayMemories Mobile, Sony).

### Surgery for open-skull cranial windows

Mice were anesthetized with 1 to 1.5% isoflurane followed by intraperitoneal injection of glycerol (0.6 g/kg) and intramuscular injection of dexamethasone (1 mg/kg). The skull was exposed, and a custom-made head-fixing metal chamber was attached with Super-Bond adhesive resin cement (Sun Medical, Shiga, Japan). The center location of cranial windows was determined by using stereotaxic coordinates in accordance with previous studies [anterior-posterior (AP) = +1.3 mm from bregma; medial-lateral (ML) = 1.2 mm from midline] (*1*, *65*, *79*). Craniotomy was bilaterally performed with a stainless steel trephine drill (Meisinger, Neuss, Germany). The double-layer glass windows were constructed with a large-diameter coverslip (no. 0, 2.3 mm diameter, Matsunami Glass, Osaka, Japan) and a small-diameter coverslip (no. 3, 2.0 mm diameter, Matsunami Glass) by using an ultraviolet-curable optical glue (NOA-61, Norland Products, NJ). The double-layer glasses were placed bilaterally on the dura and sealed with resin cement. About 1 week after the surgery, the shaping phase started as above.

### Spine dynamics observation in vivo under a two-photon microscope

Mice were anesthetized with inhalation of 0.8 to 1% isoflurane within 1 hour after the final shaping session and each training session and head-fixed on a MAG-2 head-holding device (Narishige, Tokyo, Japan) modified for our custom-made chamber. The bilateral M1s were then imaged under an upright-type Leica TCS SP8 microscope (Leica Microsystems, Wetzlar, Germany) equipped with an InSight DeepSee laser system (Spectra-Physics KK, Tokyo, Japan). GFP signal was excited with a laser of 900-nm wavelength and detected through a 525- to 550-nm filter using a 25× water-immersion objective lens [HCX IRAPO L25×, numerical aperture (NA) = 0.95]. Dendritic arborization was roughly imaged with a zoom factor of 0.75 (2048 × 2048 pixels, 0.29 μm by 0.29 μm per pixel; Z-step size, 0.85 μm), and dendritic segments derived from somata less than 400 μm deep from the pial surface (assumed to be layer 2/3 pyramidal cells) were excluded from the present analysis. High-resolution image stacks of dendritic segments were then acquired at a zoom factor of 10 (512 × 512 pixels, 0.087 μm by 0.087 μm per pixel; Z-step size, 0.85 μm, typically 20 to 30 optical sections for each). Subsequent repetitive images were acquired with the aid of blood vessels and dendritic branching patterns. In the 4-day training experiment, dendritic images were captured daily between days 0 and 4. In the 8-day training experiment, dendrites were imaged every 4 days at days 0, 4, and 8. Spines were regarded as the same among images at different training days when they were within 0.7 μm of the expected positions. Long (>1.5 μm in length from neck to tip) and thin (<0.5 μm in diameter) protrusions with no spine head (less than the spine neck diameter ×1.2) were considered as filopodia, which were excluded from the present analyses. The dendritic segments imaged were mostly located within a depth of 40 μm from the pial surface.

### Post hoc quadruple immunohistochemistry

Immediately after the final two-photon imaging (at day 4 or 8), mice were deeply anesthetized with isoflurane and transcardially perfused with 5 to 10 ml of a solution containing 250 mM sucrose and 5 mM MgCl_2_ in 0.02 M phosphate buffer (PB; pH 7.4), followed by 30 ml of 4% paraformaldehyde containing 0.2% picric acid in 0.1 M PB. Brains were then removed and postfixed for 1 hour at room temperature with the same fixative. The brains were cut into 30-μm-thick sections tangentially to the motor area on a vibrating microtome (VT1200S, Leica Microsystems). Tissue sections were stored at −30°C in cryoprotectant solution (30% glycerol and 30% ethylene glycol in 0.04 M phosphate-buffered 0.9% saline) until use.

The brain sections were washed with 0.05 M tris-buffered saline (TBS) containing 0.5% Triton X-100. The sections were then incubated for 1 week at 20°C with a mixture of rabbit anti-VGluT1 (1 μg/ml; VGluT1-Rb-Af500, Frontier Institute, Hokkaido, Japan), guinea pig anti-VGluT2 (2 μg/ml; VGluT2-GP-Af810, Frontier Institute), goat anti-Homer1 (1 μg/ml; Homer1-Go-Af1270, Frontier Institute), and chicken anti-GFP (10 μg/ml; ab13970, Abcam, Cambridge, MA) in TBS containing 0.5% Triton X-100, 10% normal donkey serum (NDS), 2% bovine serum albumin (BSA), and 0.2% sodium azide (NaN_3_). Thorough penetration of the primary antibody against VGluT1 required 1 week of incubation at 20°C (fig. S4, A to F). After several washes, the sections were incubated for 2 days at 20°C with a mixture of Alexa Fluor 568–conjugated donkey anti-rabbit (10 μg/ml; A10042, Life Technologies, Carlsbad, CA), CF405S-conjugated donkey anti–guinea pig (10 μg/ml; 20356, Biotium, Hayward, CA), Alexa Fluor 647–conjugated donkey anti-goat (10 μg/ml; A21447, Life Technologies), and Alexa Fluor 488–conjugated donkey anti-chicken (10 μg/ml; 703-545-155, Jackson ImmunoResearch Laboratories, West Grove, PA) in TBS containing 0.5% Triton X-100, 10% NDS, 2% BSA, and 0.2% NaN_3_. The brain sections were washed and then serially mounted on glass slides and coverslipped with SlowFade Gold Antifade Mountant (S36937, Thermo Fisher Scientific, Waltham, MA) or ProLong Glass Antifade Mountant (P36984, Thermo Fisher Scientific).

### Confocal laser scanning microscopy and image deconvolution

The brain sections were observed under a TCS SP8 confocal microscope (Leica Microsystems) equipped with HyD detectors. For reidentification of the dendritic branches observed in vivo, image stacks were captured using a 25× water-immersion objective lens (HCX IRAPO L25×, NA = 0.95) with the pinhole at 1.0 Airy disk unit (diameter of 48.1 μm) and a zoom factor of 0.75 (620 μm by 620 μm, 2048 × 2048 pixels, 0.303 μm by 0.303 μm per pixel in the *X* and *Y* directions; 0.445-μm interval in the *Z* direction). Alexa Fluor 488 signal was excited with a laser beam of 488-nm wavelength and observed through 500- to 550-nm emission prism windows. After the dendritic segments observed in vivo were reidentified, high-magnification image stacks were acquired using a 63× oil-immersion lens (HC PL APO CS2 63×, NA = 1.4) with a pinhole at 0.5 Airy disk unit (diameter of 50.1 μm) and a zoom factor of 3.5 (50.21 μm by 50.21 μm, 1024 × 1024 pixels, 0.049 μm by 0.049 μm per pixel in the *X* and *Y* directions; 0.129-μm interval in the *Z* direction). CF405S or Alexa Fluor 488, 568, or 647 was excited with a 405-, 488-, 552-, or 638-nm laser beam and sequentially observed through an emission prism window of 425 to 475, 500 to 550, 580 to 630, or 650 to 720 nm, respectively, in the photon-counting mode.

The acquired image stacks were deconvolved with HyVolution 2 (Leica Microsystems) with the following parameters: microscopic type, confocal; back-projected pinhole diameter, 143 nm; lens objective NA, 1.4; lens immersion refractive index, 1.518; medium refractive index, 1.515; excitation wavelength, 405, 488, 552, or 638 nm; emission wavelength, 431, 517, 603, or 665 nm; sampling *X*, *Y*, and *Z* intervals, 49.082, 49.082, and 129.2 nm; solid brick mode; using theoretical point spread function; maximum iteration number, 30; signal-to-noise ratio, 15; quality change threshold, 1 × 10^−4^.

All the presented figures were edited on the graphics software Canvas Draw (Canvas GFX Inc., Plantation, FL). Image appearance in figures was optimized uniformly in individual panels for presentation such as brightness/contrast adjustment and Gaussian filtering. The low-magnification confocal images of dendritic segments are shown in 2D projections of 3D image stacks, while the images that show spines and presynaptic and postsynaptic puncta at high magnification are presented in single focused planes.

### Somatodendritic reconstruction of GFP-labeled cells in Thy1-eGFP-M mice

Thy1-eGFP-M mice were transcardially perfused with 5 to 10 ml of a solution containing 250 mM sucrose and 5 mM MgCl_2_ in 0.02 M PB (pH 7.4), followed by 30 ml of 4% paraformaldehyde containing 0.2% picric acid in 0.1 M PB. After postfixation with the same fixative for 30 min or 1 hour, the brain tissues were sectioned into 50-μm-thick slices tangentially to the cortical surface of M1. The initial nine sections (≤~450 μm from the pia) were immunostained for GFP and Ctip2, and the deeper sections were stained in fluorescence only for Ctip2. The sections were incubated with or without chicken anti-GFP (10 μg/ml; ab13970, Abcam) in TBS containing 0.5% Triton X-100, 10% NDS, 2% BSA, 0.2% NaN_3_, and rat monoclonal antibody against Ctip2 (2 μg/ml; ab18465, Abcam). After several washes with TBS, the sections were incubated with or without Alexa Fluor 488–conjugated donkey anti-chicken (10 μg/ml; 703-545-155, Jackson ImmunoResearch Laboratories) in the same incubation solution containing Alexa Fluor 594–conjugated donkey anti-rat (10 μg/ml; A-21209, Life Technologies). The sections were serially mounted on glass slides and coverslipped with ProLong Glass Antifade Mountant (P36984, Thermo Fisher Scientific). The fluorescence signals were imaged under a TCS SP8 confocal microscope (Leica Microsystems) with a water-immersion 20× or 25× objective lens [HC PL IRAPO 20×/0.75 W (NA = 0.75) or HCX IRAPO L 25×/0.95 W 0.17 (NA = 0.95)]. Ten pairs of a Ctip2-negative pyramidal cell and its nearby Ctip2-positive cell expressing GFP in almost the same cortical depth were manually reconstructed using NeuroLucida (MBF Bioscience, Williston, VT) and analyzed quantitatively with NeuroExplorer (MBF Bioscience).

### Retrograde labeling of cells projecting to layer 1 of M1

Animals were anesthetized with an intraperitoneal injection of ketamine (100 mg/kg) and xylazine (10 mg/kg), followed by an injection of glycerol (0.6 g/kg, intraperitoneally) and dexamethasone (1 mg/kg, intramuscularly), before being placed in a stereotaxic apparatus. Craniotomy was performed on M1 with a stainless steel trephine drill (Meisinger), and the dura mater was retracted. Filter papers with a size of 1 mm by 1 mm impregnated in fluorochrome Fast Blue (Dr Illing GmbH and Co. KG, Groß-Umstadt, Hesse, Germany; 2% in distilled water) were then placed on M1 for 5 min. Five days after the surgery, the mice were fixed as described above, and the brains were cut coronally into 50-μm-thick sections. The sections were counterstained with NeuroTrace 500/525 Green Fluorescent Nissl Stain (1:100 diluted; N21480, Thermo Fisher Scientific) and propidium iodide (10 μg/ml; 29037-76; Nacalai Tesque, Kyoto, Japan) in TBS containing 0.5% Triton X-100. The fluorescence signals were captured using a TCS SP8 confocal microscope (Leica Microsystems) with 10× objective lens (HC PL APO 10×/0.40 CS, NA = 0.40).

### Virus injection and observation of M2 and thalamic axons in M1

To label M2 and thalamic axons projecting to M1, we injected AAV9.CB7.CI.mCherry.WPRE.RBG [7.36 × 10^13^ genome copies (GC)/ml; 105544, Addgene] into M2 [AP = +2.5 mm, ML = 1.2 to 1.5 mm, and dorsal-ventral (DV) = 0.8 to 1.0 mm] or the thalamus (AP = −1.0 mm, ML = 1.1 to 1.2 mm, and DV = 3.5 to 4.0 mm) of C57BL/6J or Thy1-eGFP-M mice. Mice were fixed 1 week after the injections, and the brains were cut coronally or tangentially into 30- or 50-μm-thick sections as described above.

The virus-injected brain sections of C57BL/6J mice were triple-immunostained for mCherry, VGluT1, and VGluT2. The 30-μm-thick coronal sections were incubated at 20°C for 1 week in TBS containing 0.5% Triton X-100, 10% NDS, 2% BSA, and 0.2% NaN_3_ with primary antibodies rabbit anti-DsRed (1:1000 diluted; 632496, Clontech, Mountain View, CA), goat anti-VGluT1 (1 μg/ml; VGluT1-Go-Af310, Frontier Institute), and guinea pig anti-VGluT2 (2 μg/ml; VGluT2-GP-Af810, Frontier Institute). After washes, the sections were incubated in the same solution with secondary antibodies Alexa Fluor 594–conjugated donkey anti-rabbit (10 μg/ml; A-21207, Life Technologies), Alexa Fluor 488–conjugated donkey anti-goat (10 μg/ml; A-11055, Life Technologies), and CF405S-conjugated donkey anti–guinea pig (10 μg/ml; 20356, Biotium). The sections were observed under the confocal microscope at high magnification with a water-immersion 63× objective lens (HC PL APO 63×/1.20 W CORR CS2, NA = 1.2) at a zoom factor of 3.5, followed by image deconvolution as described above. The colocalization rates of signals immunoreactive for VGluTs with axon terminal–like structure (“bouton”) were quantified using the Fiji software (https://imagej.net/Fiji). The axonal varicosities were regarded as boutons when their size was more than twofold larger than the thickness of the intervaricose fibers.

The brain sections obtained from Thy1-eGFP-M mice were triple-immunostained for GFP, mCherry, and Homer1. The tissues were incubated for 2 days at 20°C with chicken anti-GFP (10 μg/ml; ab13970, Abcam), mouse anti-mCherry (1 μg/ml; 632543, Clontech, Mountain View, CA), and goat anti-Homer1 (1 μg/ml; Homer1-Go-Af1270, Frontier Institute) in TBS containing 0.5% Triton X-100, 10% NDS, 2% BSA, and 0.2% NaN_3_. The sections were then washed and incubated with a mixture of Alexa Fluor 488–conjugated donkey anti-chicken (10 μg/ml; 703-545-155, Jackson ImmunoResearch Laboratories), Alexa Fluor 594–conjugated donkey anti-mouse (10 μg/ml; A-21203, Life Technologies), and CF405S-conjugated donkey anti-goat (10 μg/ml; 20416, Biotium) in the same incubation solution. The sections were observed at high magnification under the confocal microscope as described above. Close apposition of M2 or thalamic axon varicosities on Homer1-immunoreactive puncta in spines was regarded as putative excitatory synapses. The numbers of putative synaptic inputs were counted in both apical tufts and basal dendrites of single pyramidal cells.

### Chemogenetic inactivation during motor learning

For selective inactivation of M1p M2 or thalamic neurons, AAV8- or AAV9-hSyn-DIO-hM4D(Gi)-mCherry (2.9 or 2.3 × 10^13^ GC/ml; 44362, Addgene) was bilaterally injected into M2 (AP = +2.5 mm, ML = 1.2 to 1.5 mm, and DV = 0.8 to 1.0 mm) or the thalamus (AP = −1.0 mm, ML = 1.1 to 1.2 mm, and DV = 3.5 to 4.0 mm) of 4-week-old C57BL/6J male mice (the Jackson Laboratory, Bar Harbor, ME), with the injection of AAVrg-hSyn-Cre-WPRE-hGH (1.57 × 10^13^ GC/ml; 105553, Addgene) into M1 (AP = +1.3 mm, ML = 1.2 mm, and DV = 0.2 mm). For the control experiments, AAV8- or AAV9-hSyn-DIO-mCherry (2.3 × 10^13^ or 2.1 × 10^13^ GC/ml; 50459, Addgene) was injected into M2 or the thalamus instead of AAV-hSyn-DIO-hM4D(Gi)-mCherry.

Shaping started 4 weeks after the virus injection, followed by training of the single-seed reaching task. CNO (5 mg/kg of body weight; C0832, Sigma-Aldrich) was intraperitoneally injected 30 min before every training from days 1 to 8. Two-photon imaging with chemogenetic silencing (Fig. 9) was performed at days 0, 4, and 8 as described above.

Virus infection was confirmed by mCherry fluorescence expression in the fixed brain sections after all the training sessions. Cortical lamination and thalamic nuclei were determined with fluorescence Nissl counterstaining, and mCherry fluorescence was enhanced by immunofluorescence staining. After washing with 0.05 M TBS containing 0.5% Triton X-100, 50-μm-thick brain sections were incubated for 2 days at 20°C in TBS containing 0.5% Triton X-100, 10% NDS, 2% BSA, and 0.2% NaN_3_ with a primary antibody against DsRed (rabbit antibody, 632496, Clontech). After several washes, the sections were incubated overnight at 20°C with Alexa Fluor 568–conjugated donkey anti-rabbit (10 μg/ml; A10042, Life Technologies) in the same incubation solution. The brain sections were lastly counterstained with NeuroTrace 435/455 Blue Fluorescent Nissl Stain (1:100 diluted; N21479, Thermo Fisher Scientific) in 0.05 M TBS containing 0.5% Triton X-100. The tissues were mounted and coverslipped as described above. Although the original literature of AAVrg reported inefficient retrograde transduction of GFP in motor-thalamic neurons that project to M1 (*80*), our dual injection of AAVs worked well so that both cell bodies in the motor thalamic nuclei and axon fibers in M1 were clearly labeled with mCherry signal, indicating sufficient expression of hM4D(Gi) in TC neurons (Fig. 4F and fig. S10B).

For pathway-selective silencing of M2 → M1 projection, CNO was locally injected into cM1. Four weeks after the virus injection as above, the custom-made head-fixing metal chamber was attached with Super-Bond adhesive resin cement (Sun Medical) under brief anesthesia with inhalation of isoflurane, and small holes were made over the skull bilaterally on M1 (AP = +1.3 mm and ML = 1.2 mm). The holes were covered with silicone caps (Kwik-Cast silicone sealant, World Precision Instruments, Sarasota, FL). Shaping sessions were performed without CNO application. Individual training sessions started 5 min after local injection of 200 nl of 10 μM CNO into cM1 through glass pipettes. The CNO-injected site was marked by injection of 2% Chicago Sky Blue 6B (C8679, Sigma-Aldrich) in 200 nl of saline into M1 after the final training session at day 8. Since Chicago Sky Blue 6B showed red fluorescence signal, mCherry fluorescence was immunolabeled with Alexa Fluor 488. The brains were fixed as described above after the injection of Chicago Sky Blue 6B and cut into 50-μm-thick sections. The tissue sections were incubated with a primary antibody against DsRed (rabbit antibody, 632496, Clontech), followed by incubation with Alexa Fluor 488–conjugated donkey anti-rabbit antibody (10 μg/ml; A21206, Life Technologies). Cortical lamination was determined by counterstaining with NeuroTrace 435/455 Blue Fluorescent Nissl Stain as above.

To confirm the effect of chemogenetic inactivation on postsynaptic neurons in M1, we electrophysiologically recorded the response of M1 neuron activity evoked by optogenetic stimulation of presynaptic neurons (Fig. 4B and figs. S9 and S10). We injected a mixture of AAV8-EF1a-DIO-hChR2(H134R)-mCherry-WPRE-hGHpA (Addgene, 20297) and AAV8-hSyn-DIO-hM4D(Gi)-mCherry into M2 (AP = +2.5 mm, ML = 1.2 to 1.5 mm, and DV = 0.8 to 1.0 mm) or the thalamus (AP = −1.0 mm, ML = 1.1 to 1.2 mm, and DV = 3.5 to 4.0 mm) of C57BL/6 mice in addition to the injection of AAVrg-hSyn-Cre-WPRE-hGH into M1 (AP = +1.3 mm, ML = 1.2 mm, and DV = 0.3 mm). Four weeks after the injection, we anesthetized animals with isoflurane (~2%) and then urethane (0.05 mg/kg of body weight) and stabilized their heads in the stereotaxic instrument. Lidocaine [1% (w/v), Braun, Melsungen, Germany] was injected around the surgical site. Body temperature was maintained at ~36°C by a heating pad, and the depth of anesthesia was monitored throughout the experiment. For electrophysiological recording of M2 → M1 innervation, we made craniotomies above M1 and M2 and perpendicularly inserted a linear array of 16 electrodes (A1 × 16-5 mm-100-177-A16, NeuroNexus, Ann Arbor, MI) as the uppermost electrodes were positioned at 100 μm below the pial surface. A 473-nm blue light-emitting diode was delivered to M2 using an optic fiber with a 400-μm core (#57-063, Edmund Optics GmbH, Mainz, Germany) placed on the pial surface. Recording of thalamic innervation in M1 was performed with a glass pipette positioned at 20 to 50 μm from the pial surface where TC fibers were largely localized, with blue-light illumination of axon fibers in M1. Photostimulation was conducted with 0.5 Hz, and the duration was 20 or 100 ms. Electrical activity was band-pass filtered at 1 to 9 kHz, digitized at 10 kHz, and amplified by the ERP-27 system and Cheetah software (Neuralynx, Tucson, AZ). As illustrated in Fig. 4B and figs. S9E and S10C, the amplitude of evoked potentials was measured between peaks (*81*).

### CLEM with ATUM-SEM

Synapse formation at the putative synaptic input sites determined by post hoc immunohistochemistry and the correlation between spine head size and fluorescence in spine heads were confirmed by CLEM with ATUM-SEM (Automated tape-collecting ultramicrotome combined with scanning electron microscopy). Immediately after the final two-photon imaging session at day 8, mice were fixed as described above under deep anesthesia. Perfused fixative additionally contained 1% glutaraldehyde and was postfixed in the body for 1 hour at room temperature. The brain surface tangential to the motor area was cut into a ~150-μm-thick section on a vibrating microtome. The tissue was stored at −30°C in cryoprotectant solution until use.

The section was counterstained in fluorescence for 2 hours at 4°C with 4′,6-diamidino-2-phenylindole (5 μg/ml; DAPI; 10236276001, Roche, Basel, Switzerland) and DyLight 594–labeled *Lycopersicon esculentum* (tomato) lectin (20 μg/ml; DL-1177, Vector Laboratories, Burlingame, CA) in 0.05 M TBS containing 1% BSA. DAPI and DyLight 594 signals visualized nuclei and blood vessels, respectively, in different fluorescence from GFP for landmarks of the following CLEM. The section was mounted upside down on a glass-bottom dish with SlowFade Gold Antifade Mountant and observed under an inverted-type TCS SP8 confocal microscope. Image stacks were obtained using a 25× water-immersion objective lens (HCX IRAPO L25×, NA = 0.95) with the pinhole at 1.0 Airy disk unit (diameter of 48.1 μm) and a zoom factor of 0.75 (620 μm by 620 μm, 2048 × 2048 pixels, 0.303 μm by 0.303 μm per pixel in the *X* and *Y* directions; 0.502-μm interval in the *Z* direction). DAPI, GFP, and DyLight 594 signals were excited with 405-, 488-, and 552-nm laser beams and sequentially observed through emission prism windows of 410 to 489, 500 to 550, and 600 to 700 nm, respectively.

After the confocal microscopy, the tissue was processed for EM observation as previously reported (*47*) with slight modification. The following procedures were performed at room temperature unless otherwise stated. The tissue was washed with 0.1 M PB followed by washes with 0.1 M cacodylate buffer (pH 7.4). The section was then postfixed in 1.5% potassium ferrocyanide and 2% osmium tetroxide (OsO_4_) in 0.1 M cacodylate buffer at 4°C for 1 hour. After postfixation, the tissue was washed in ultrapure water (Milli-Q Reference water purification system, Merck Millipore, Burlington, MA) and subsequently stained with 1% thiocarbohydrazide for 20 min, followed by washes with ultrapure water. The section was again postfixed in 2% OsO_4_ for 30 min and, after washing with ultrapure water, stained overnight at 4°C with 1% uranyl acetate. The tissue washed with ultrapure water was processed with modified Walton’s en bloc lead aspartate staining (*82*). Lead aspartate solution was prepared by dissolving 0.066 g of lead nitrate in 10 ml of 0.03 M aspartic acid, pH adjusted to 5.0 with 1 N potassium hydroxide, and kept at 50°C until dissolved. The tissue was stained with the lead aspartate solution at 50°C for 2 hours, followed by washes with ultrapure water. The section was then dehydrated in graded dilutions of ethanol and embedded on silicon-coated glass slides in epoxy resin (Durcupan ACM; Sigma-Aldrich, St. Louis, MO). The sample was polymerized at 70°C for 3 days.

The embedded tissue was serially resectioned into 50-nm-thick ultrathin sections and collected using an automated tape-collecting ultramicrotome (ATUMtome; Boeckeler Instruments Inc., Tucson, AZ) on a plasma-hydrophilized carbon nanotube–coated polyethylene terephthalate tape (CNT-PET tape) (*47*). The following scanning EM observation was described previously (*47*). Briefly, serial ultrathin sections on tape were cut into strips and mounted in order on 10 cm silicon wafers with double-sided adhesive conductive tape. The conductive surface of the CNT-PET tape was grounded to the wafer with copper foil tape. The serial EM images were obtained using a backscattered-electron detector of field-emission scanning electron microscopy (Sigma-Aldrich, Carl-Zeiss Microscopy, Oberkochen, Germany) with a guide of Atlas 5 (Fibics Incorporated, Ottawa, Canada). EM images were captured at a resolution of 5 nm by 5 nm per pixel in the *X* and *Y* directions.

We obtained EM image stacks of two adjacent regions separately from one brain sample [approximately 200 μm by 200 μm in *XY*, 662 sections of 50 nm thickness (a total of 33.1 μm in depth); 100 μm by 150 μm in *XY*, 496 sections (a total of 24.8 μm in depth)]. Tiled images in a single plane were stitched, and serial mosaic images were aligned on TrakEM2 (https://imagej.net/TrakEM2). The 3D EM images were downscaled to a resolution of 10 nm by 10 nm per pixel in the *XY* direction and imported to the segmentation software VAST Lite (https://lichtman.rc.fas.harvard.edu/vast/). After the dendritic segments observed in the two-photon images were reidentified in the confocal fluorescence images, nuclei and blood vessels labeled in fluorescence were found in the EM image stacks. On the basis of such landmarks, the dendritic segments were distinguished from surrounding neuronal and non-neuronal microstructures and manually reconstructed on the VAST Lite software. Spine and bouton volumes of reconstructed data from the EM images were measured on the VastTools MATLAB scripts for VAST Lite.

### Synapse confirmation of putative synaptic input sites with EM observation

For EM observation of fluorescently identified putative synaptic input sites (fig. S4, G to L), a Thy1-eGFP-M mouse was perfused with 1% glutaraldehyde–containing fixative as above and cut into a ~100-μm-thick section on a vibrating microtome. The section was immunostained for GFP, VGluT1, and Homer1 and observed under a confocal microscope as described above to identify putative synaptic input sites where VGluT1- and Homer1-positive puncta were closely apposed on GFP-labeled spines. The tissue processed for EM observation as above was cut into 50-nm-thick ultrathin sections and serially mounted on a CNT-PET tape. The serial EM images were obtained using field-emission scanning electron microscopy (Regulus 8240, Hitachi High-Tech, Tokyo, Japan) controlled by the Auto Capture for Array Tomography software (Hitachi High-Tech). EM images were captured at a resolution of 4.96 nm by 4.96 nm per pixel in the *X* and *Y* directions. Following EM, image alignment and 3D reconstruction were performed as described above.

### Spine number counting and data analysis

Spine dynamics were analyzed manually on Fiji. The spine location of the identical dendritic segments (typically 45 to 60 μm in length; cM1 and iM1 in the 4-day experiment, *n* = 171 and 175 dendritic segments, respectively; in the 8-day experiment, *n* = 91 and 104 segments, respectively) among several imaging sessions was identified in 3D image stacks. A spine within 0.7 μm of its expected position from the previous imaging session was regarded as the same spine. Spines observable throughout all images were determined as “stable spines”; spines that were absent in the image at day 0 and that appeared in the subsequent images were categorized as “newly formed spines”; spines observable at day 0 that disappeared in the subsequent imaging sessions were defined as “eliminated spines.” Spine formation and elimination rates were normalized to the initial image at day 0. The dendritic images shown in figures were single focused planes modified for presentation through brightness/contrast adjustment and Gaussian blur filtering uniformly in individual images. Filopodia, which are long thin structures without spine heads or Homer1 immunoreactivity, were excluded from the present analysis.

Quadruple-fluorescence images were also analyzed on Fiji. The image stacks in 3D were observed, and contacts of presynaptic and postsynaptic puncta on spines were regarded as synaptic input sites. Dendrites and presynaptic and postsynaptic markers were individually pseudocolored, and each signal could be visualized and hidden by the color channel tool on Fiji. Spines were identified by comparing the two-photon and confocal images, followed by confirmation of Homer1 immunoreactivity in spine heads. Presynaptic markers, VGluT1 and VGluT2, were then visualized, and presynaptic puncta were classified into CC or TC axons. Some presynaptic VGluT-positive puncta made contacts on Homer1 signals in the *Z* axis. When fluorescence intensities of individual boutons were normalized to the maximum intensity in the same captured images, putative presynaptic boutons had peaks of fluorescence intensity near the peaks of Homer1 signals, with more than 0.15 arbitrary units (a.u.) (the maximum intensities of individual signals in the optical sections were normalized to 1 a.u.; fig. S3). When the fluorescence signals of GFP, Homer1, and VGluTs were placed in this order, the contact sites were regarded as putative synapses. In some cases where the presynaptic puncta on Homer1 in single spines were indistinguishable and both VGluT1 and VGluT2 signals made contacts, the spines were classified as “CC + TC.” In the other cases where no presynaptic axons were located on Homer1 signals, the spines were classified as “none.” Spines are occasionally located between brain sections and sometimes lost, but 97.2% (4-day training) and 99.1% (8-day training) of spines observed in vivo at the final imaging sessions could be reidentified in post hoc immunostained sections.

Spine fluorescence intensity was measured using Fiji as well. The outlines of spine heads were determined by the images processed with the Kuwahara filter built in Fiji for edge detection (fig. S14). The mean fluorescence in the dotted line was measured at the optical section with the maximum intensity. The mean fluorescence intensity in the spine heads of the original images was normalized by subtracting the background fluorescence, and then divided by the shaft intensity (minus background intensity) within 2 μm centered on the spine necks.

### Statistical analysis

Values are shown as means ± SEM. Statistics were performed on Prism 8 (version 8.2; GraphPad Software, San Diego, CA). Paired samples were compared using two-tailed Wilcoxon matched-pairs signed-rank test. Unpaired samples were compared with two-tailed Mann-Whitney test. Pearson correlation coefficient was calculated with linear regression analysis of two numeric variables. Statistical significances of the differences between two regression slopes were determined by ANCOVA-based regression comparison. Matched multiple populations were compared with Friedman test followed by post hoc Dunn’s multiple comparisons test. Unmatched multiple populations were compared with Kruskal-Wallis test followed by post hoc Dunn’s multiple comparisons test. Two-sided Fisher’s exact test or chi-square test was performed for comparison between categorical data. ΔFluorescence intensity was compared with Kolmogorov-Smirnov test.

## Notes

View/request a protocol for this paper from *Bio-protocol*.
