# Supplementary material for: Presynaptic supervision of cortical spine dynamics in motor learning
Source: Sci Adv. 2022 Jul 27;8(30):eabm0531. doi: 10.1126/sciadv.abm0531 (PMC9328689; doi:10.1126/sciadv.abm0531)
Supplement: Supplementary file 1 — Supplementary Text Table S1 Figs. S1 to S15 [file sciadv.abm0531_sm.pdf]

Science Advances
AAAS

Supplementary Materials for

Presynaptic supervision of cortical spine dynamics in motor learning

Jaerin Sohn *et al.*

Corresponding author: Yoshiyuki Kubota, yoshiy@nips.ac.jp

Sci. Adv. **8**, eabm0531 (2022)
DOI: 10.1126/sciadv.abm0531

The PDF file includes:

Supplementary Text
Table S1
Figs. S1 to S15
Legends for movies S1 and S2

Other Supplementary Material for this manuscript includes the following:

Movies S1 and S2

Supplementary Text

Calculation of spine formation/elimination activity

Spine formation activity is total numbers per length [µm] of spines formed during 4-day training no matter whether the new spines were observable at day 4 or eliminated by day 4. Spine elimination activity is total number per length of spines eliminated during 4-day training including transiently-formed spines. Spine formation and elimination activities are defined as following:

$$\textit{Spine formation activity}\ (/\mu\text{m}) = \frac{n_{tran} + n_{new} - 1}{L_{segment}}$$

*at the dendritic segments with new TCi spines

$$\textit{Spine formation activity}\ (/\mu\text{m}) = \frac{n_{tran} + n_{new}}{L_{segment}}$$

*at the dendritic segments without new TCi spines

$$\textit{Spine elimination activity}\ (/\mu\text{m}) = \frac{n_{tran} + n_{elim}}{L_{segment}}$$

where n_{tran} is the number of spines formed from day 1 to day 3 that disappeared by day 4, and n_{new} is the number of newly-formed spines observed at day 4. Also, n_{elim} is the number of spines observed at day 0 but eliminated by day 4. L_{segment} is the length (µm) of dendritic segments without bifurcation in the region of interest (51.4 ± 0.4 µm, mean ± SEM; n = 347).

Survival-rate estimation of CCi and TCi spines during day 4 to day 8

To estimate the proportion of spines maintained until at day 8 after spinogenesis by day 4, we assumed that the proportion of new CCi and TCi spines at day 4 in the 8-day experiment (Figs. 6 and 7) was consistent with the result in the 4-day examination (Figs. 1 and 2). The calculation formula is as follows:

$$\textit{CCi spine survival rate}\ (\%) = \frac{F^{CCi}_{Per}}{(F^{All}_{Tran} + F^{All}_{Per}) \times P^{CCi}_{d4}} \times 100$$

$$\textit{TCi spine survival rate}\ (\%) = \frac{F^{TCi}_{Per}}{(F^{All}_{Tran} + F^{All}_{Per}) \times P^{TCi}_{d4}} \times 100$$

where F^{CCi}_{Per} or F^{TCi}_{Per} is the frequency (%) of persistently-formed CCi or TCi spines at day 8 (Fig. 7, D and E), and F^{All}_{Tran} or F^{All}_{Per} is the frequency (%) of transiently- or persistently-formed spines, respectively, in the 8-day experiment (Fig. 6B). P^{CCi}_{d4} or P^{TCi}_{d4} is the proportion of new CCi or TCi spines observed at day 4 in the mice with more than 10% increase of success rate (success rate slope > 3.3) during the 4-day training (Fig. 2, C to F). The values in Table S1 are used for calculation.

Supplementary Table

	Value	Note
F_{Tran}^{All} (%)	5.83	Mean formation rate of all transiently-formed spines (8-day experiment)
F_{Per}^{All} (%)	4.53	Mean formation rate of all persistently-formed spines (8-day experiment)
F_{Per}^{CCi} (%)	1.22	Mean formation rate of persistently-formed CCi spines (8-day experiment)
F_{Per}^{TCi} (%)	3.10	Mean formation rate of persistently-formed TCi spines (8-day experiment)
P_{d4}^{CCi}	0.654	Proportion of CCi spines in spines formed by day 4 (4-day experiment)
P_{d4}^{TCi}	0.333	Proportion of TCi spines in spines formed by day 4 (4-day experiment)

Table S1. Frequency and proportion of new spines formed during 4 or 8 days of the learning for calculation of CCi and TCi spine survival rate.

The values F_{Tran}^{All}, F_{Per}^{All}, F_{Per}^{CCi}, F_{Per}^{TCi} are from Figs. 6B and 7E. The proportions of new CCi and TCi spines in all spines formed by day 4, P_{d4}^{CCi} and P_{d4}^{TCi}, are from Fig. 2D.

Supplementary Figures

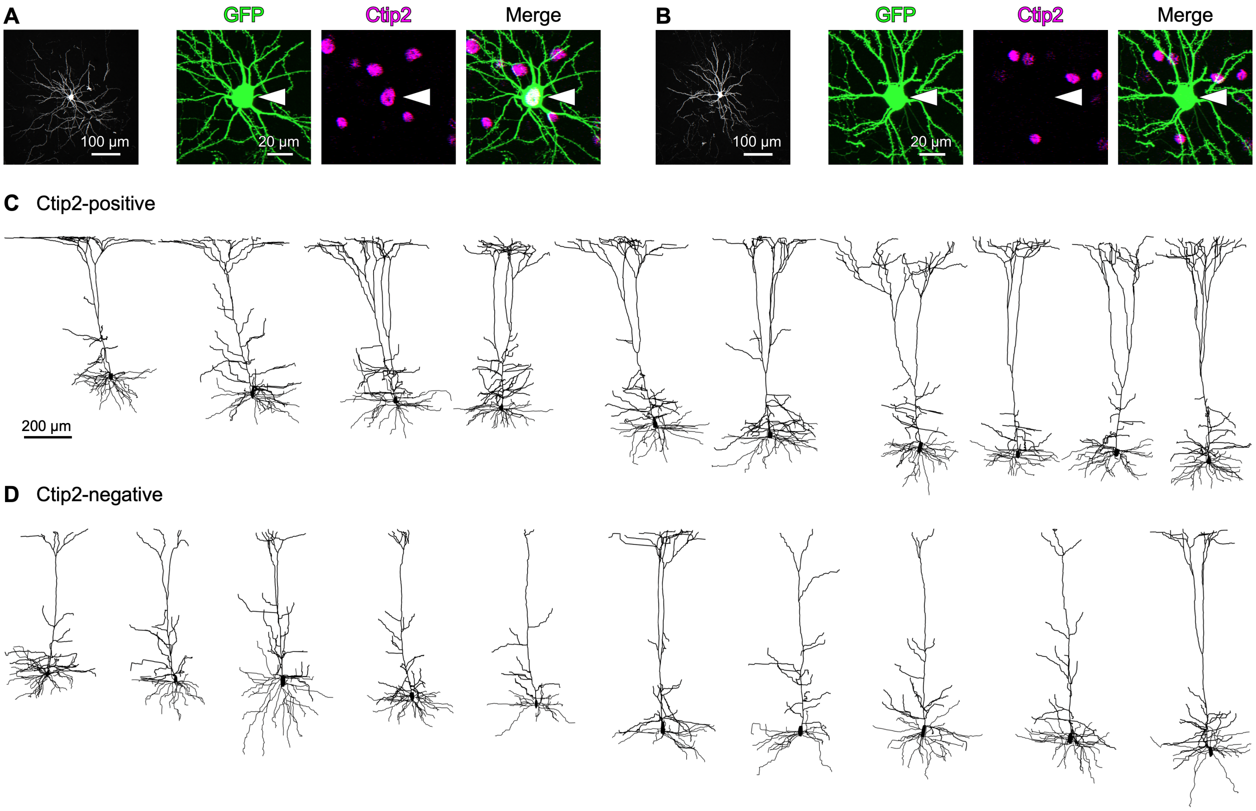

Fig. S1. Characterization of sparsely-distributed GFP-labeled cells in Thy1-eGFP-M mouse line.

(**A** and **B**) GFP-labeled layer 5 pyramidal cells positive (**A**) or negative (**B**) for Ctip2 in Thy1-eGFP-M mice (tangential sections). Ctip2 is expressed in 89.5 ± 2.4% (mean ± SEM; 260/289, n = 10 mice) of GFP-labeled cells.

(**C** and **D**) Somatodendritic morphologies of Ctip2-positive (**C**) and Ctip2-negative (**D**) cells (reconstructed vertically). Total lengths per Ctip2-positive and Ctip2-negative cells of the apical tuft within a depth of 40 µm from the cortical surface are 2.26 ± 0.24 mm/cell and 0.49 ± 0.130 mm/cell (mean ± SEM), respectively.

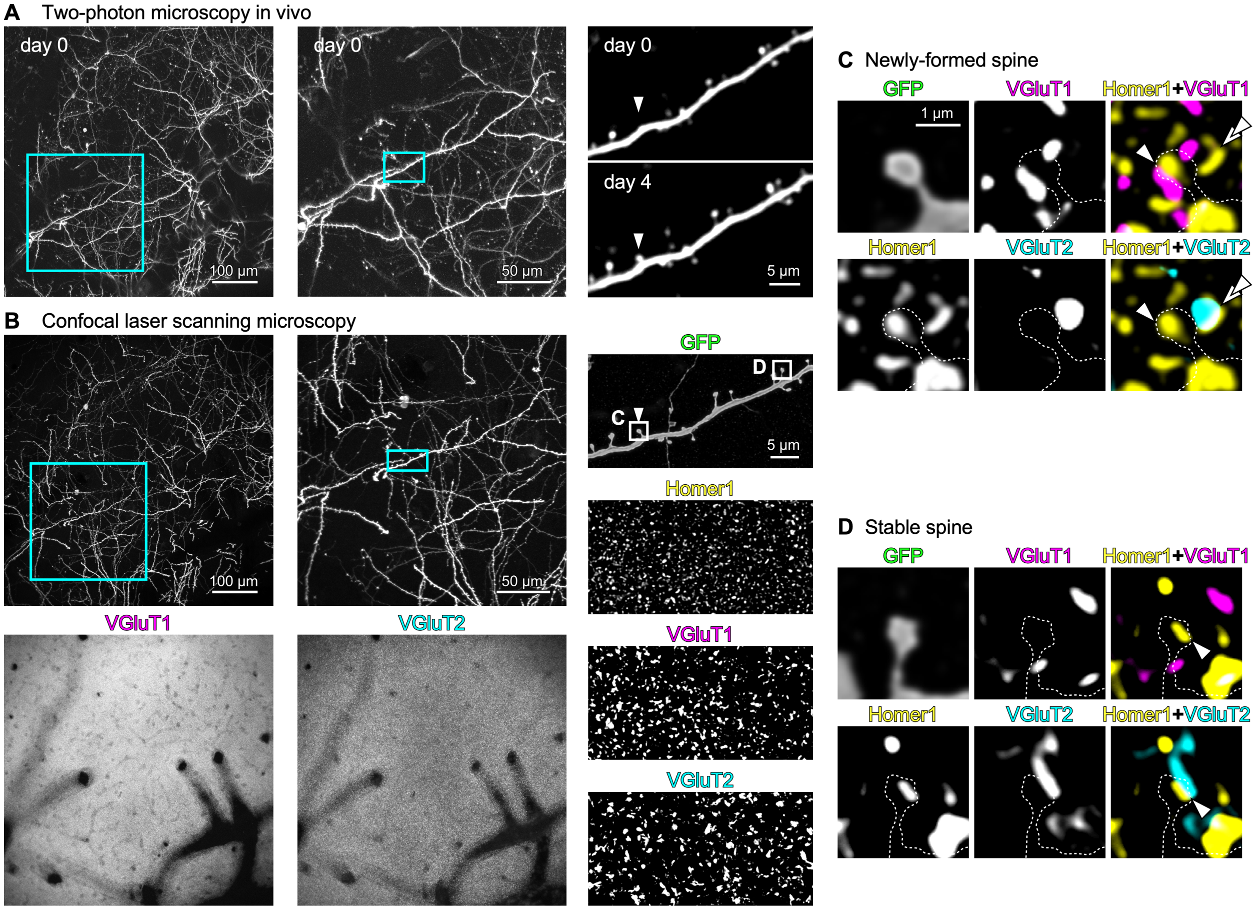

Fig. S2. Post hoc immunohistochemistry after two-photon observation of spine dynamics in vivo.

(**A**) Two-photon images in vivo during 4-day training. (Left, middle) Low-magnification images of M1. (Right) High-magnification images at day 0 and day 4. An arrowhead indicates a newly-formed spine.

(**B**) Confocal images with post hoc immunostaining for GFP, Homer1, VGluT1 and VGluT2 corresponding to (**A**). The dendrites observed in vivo were identified with the aid of blood-vessel and dendrite branching patterns.

(**C** and **D**) Newly-formed (**C**) and stable (**D**) spines were observed in quadruple fluorescence images. Arrowheads indicate putative synaptic input sites. A double-arrowhead indicate a TC axon terminal close to the new spine that innervates a different target.

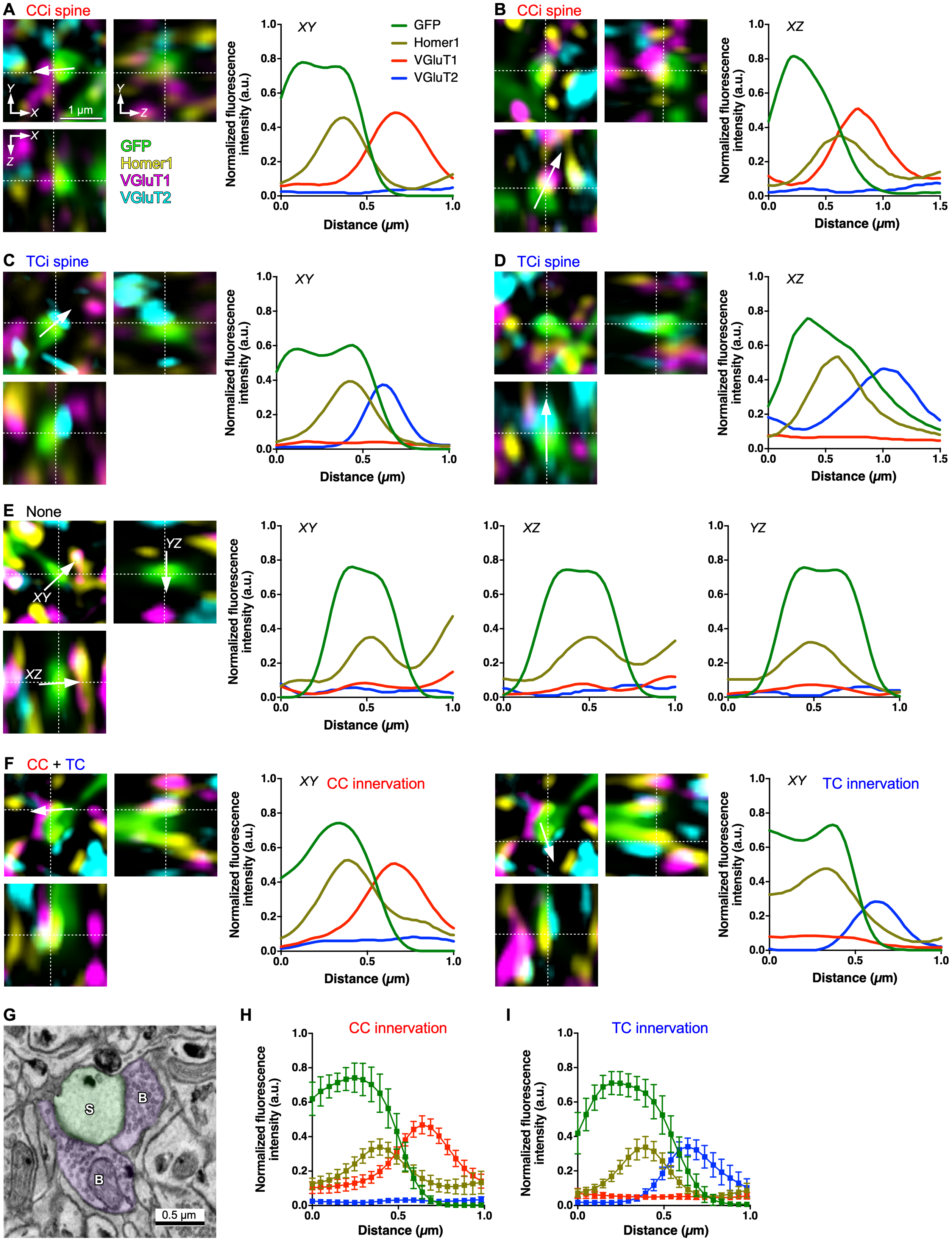

A CCi spine
1 µm
GFP
Homer1
VGluT1
VGluT2
XY
Normalized fluorescence intensity (a.u.)
Distance (µm)
B CCi spine
XZ
C TCi spine
D TCi spine
E None
XY
XZ
YZ
F CC + TC
CC innervation
TC innervation
G
S
B
0.5 µm
H CC innervation
I TC innervation

Fig. S3. Characterization of presynaptic cell types innervating individual spines in confocal images with post-hoc immunohistochemistry.

(**A**) (Left) An orthogonal view of quadruple immunostaining for GFP (green), Homer1 (yellow), VGluT1 (magenta) and VGluT2 (cyan) in the *XY*, *YZ*, *ZX* planes. (Right) Fluorescence intensities along the arrow in the left panel normalized to the maximum intensities of individual signals in the optical section (maximum intensities are normalized to 1 a.u.). This example shows that a VGluT1-immunoreactive punctum makes a close contact on a Homer1 signal in a GFP-labeled spine head, categorized as a 'CCi spine'.
(**B**) An example of a CCi spine with a putative synaptic contact in the *Z* direction. VGluT1 immunoreactivity is not observable in the *X*Y plane, while a VGluT1-positive punctum is closely apposed to a Homer1 signal in a GFP-labeled spine head in the *YZ*, *ZX* planes.
(**C**) An example of a 'TCi spine'. A putative synaptic contact is observable in the *X*Y plane.
(**D**) An example of a TCi spine with a putative synaptic contact in the *Z* direction.
(**E**) An example image of a GFP-labeled spine head without visible apposition of VGluT signals, categorized as 'None'.
(**F**) An example image with two appositions of both VGluT1 and VGluT2 signals on a single spine head, categorized as 'CC+TC'.
(**G**) An example EM image of a dually-innervated spine. As dual innervations on a single spine head are observable by confocal microscopy like the image in (**F**), EM occasionally identifies spine heads with two synaptic structures. S, spine; B, bouton.
(**H** and **I**) Mean fluorescence intensities of individual signals in CCi (**H**; $n = 23$ spines from 9 mice) and TCi (**I**; $n = 21$ spines from 9 mice) spines (mean ± 95% confidence interval). The apposed signals of VGluTs show clear peaks with more than 0.15 a.u. in the vicinity of Homer1 signal peaks.

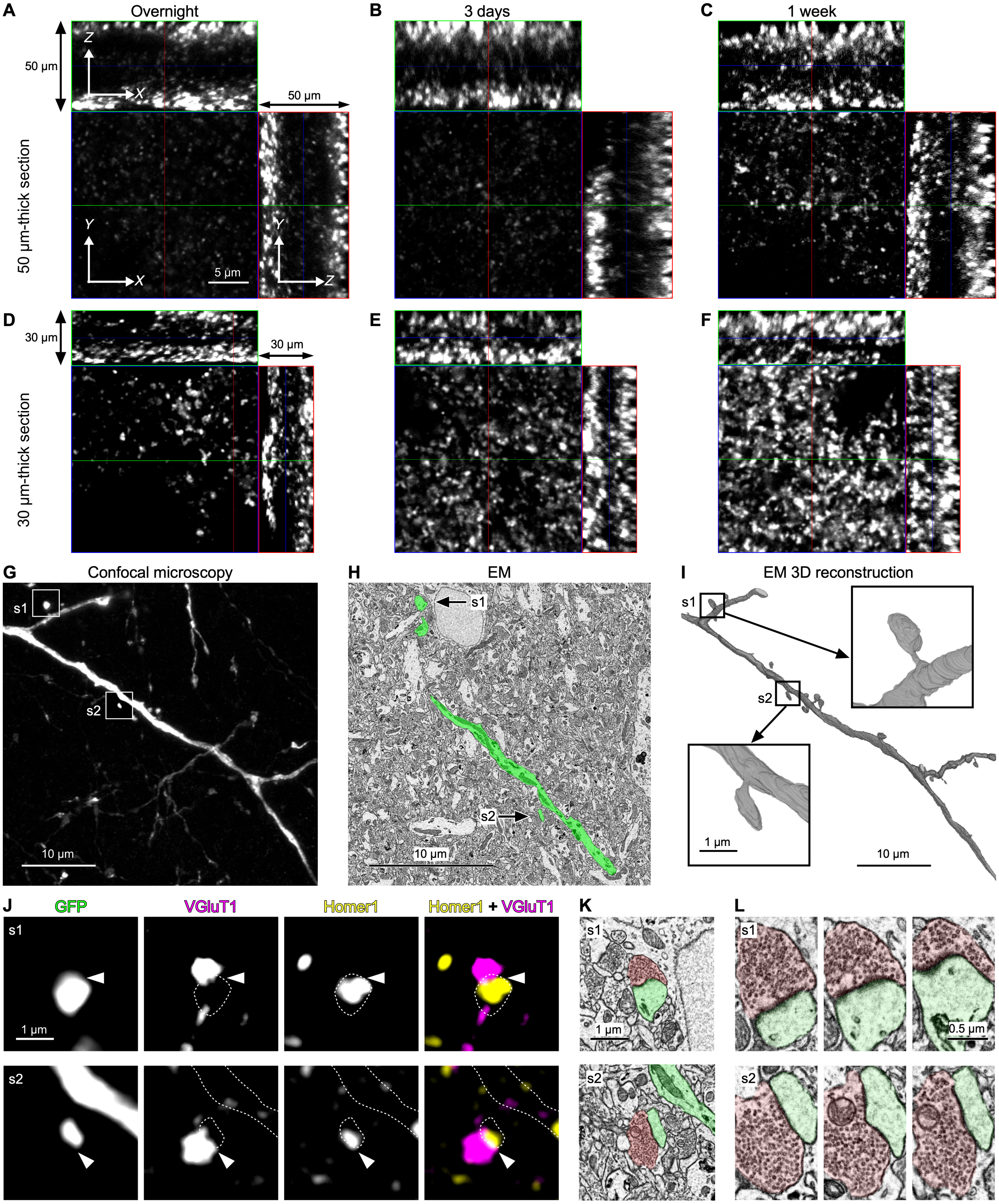

Fig. S4. **Contact sites of presynaptic and postsynaptic puncta form synapse**.

(**A** to **F**) Orthogonal views of immunostained 50- (**A** to **C**) or 30-µm-thick (**D** to **F**) sections for VGluT1 incubated overnight (**A** and **D**), for 3 days (**B** and **E**) or for 1 week (**C** and **F**). The

antibody completely penetrates into the inner part of the sections when 30-μm-thick sections are incubated for 1 week at 20 ºC (**F**).
(**G** to **I**) Correlated confocal and electron microscopy. A dendritic segment (**G**) can be observed in an EM image (**H**) and three-dimensionally reconstructed (**I**). The dendritic segment corresponding to (**G**) is labeled with green in (**H**).
(**J**) Magnified fluorescence images of spines in (**G**) with immunolabeling for VGluT1 (magenta) and Homer1 (yellow). Arrowheads indicate contact sites of VGluT1 and Homer1 on GFP-labeled spines.
(**K** and **L**) Synaptic structure is actually observable in EM images at putative synaptic contact sites in (**J**). Dendrites and boutons are shown in light green and red, respectively. High-magnification EM images of spines (s1 and s2) in different ultrathin sections are displayed in (**L**).

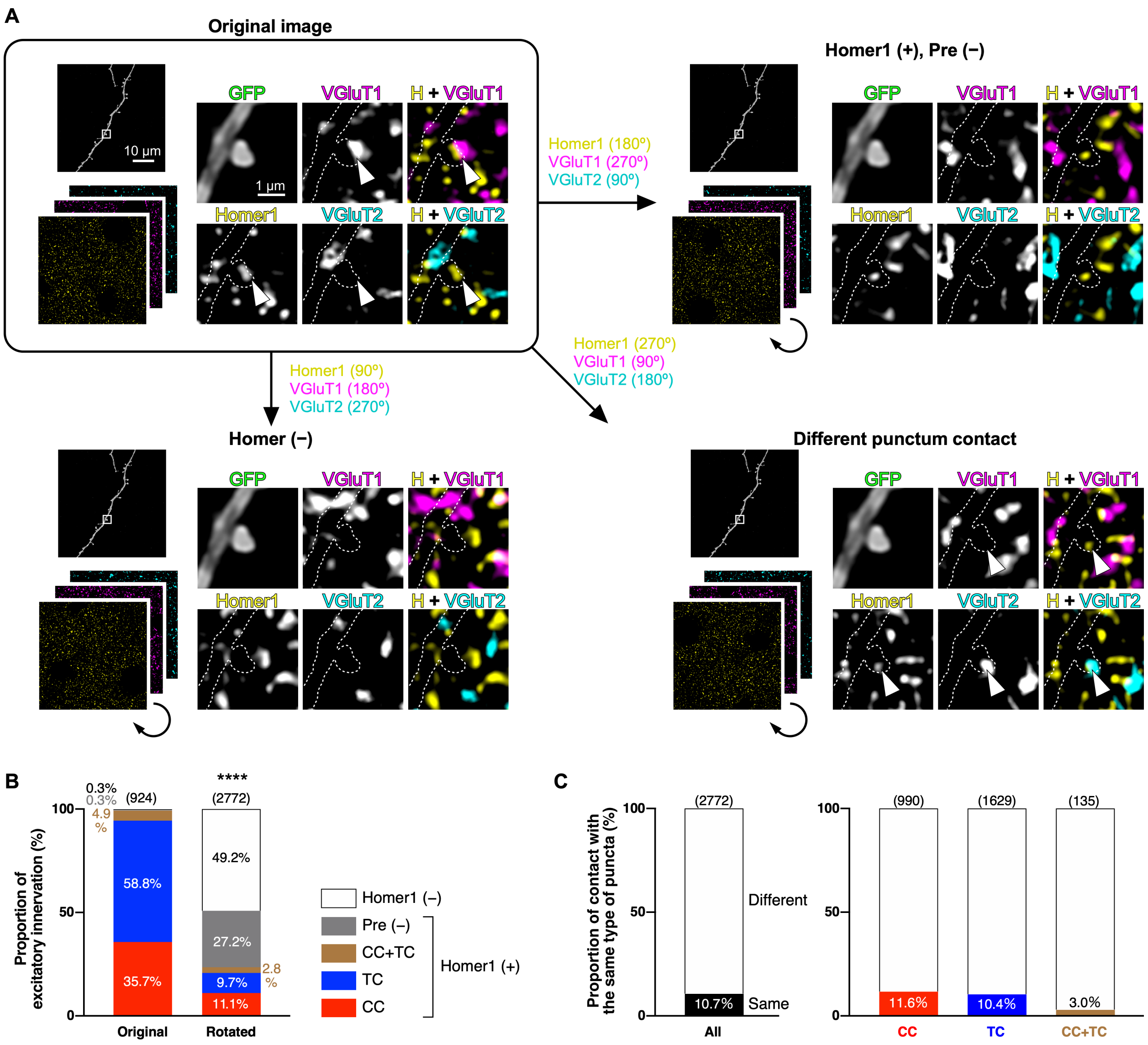

Fig. S5. Contacts of VGluT signals on Homer1 immunoreactivity in spines occurred at a substantially higher rate than chance.

(**A**) Quantification of VGluT and Homer1 contacts in spines after rotation of Homer1 and VGluT images. The number of close contacts after rotation of Homer1, VGluT1 and VGluT2 immunofluorescence images (90º, 180º and 270º) was counted (n = 90 dendritic segments from 9 mice). After the image rotations of the original image (upper left; a CCi spine), the spine does not include Homer1 signals (bottom left; "Homer1(−)"), it contains a Homer1 signal without contacts of VGluT signals (upper right; "Homer1(+), pre (−)"), or a Homer1 signal in spine is contacted by a VGluT2 punctum (bottom right; "Different punctum contact").
(**B**) Proportion of pseudo-contacts after image rotation. About a half of spines did not overlap with Homer1 immunoreactive puncta ("Homer1(−)"). Less than a quarter of them contained Homer1 immunoreactive puncta contacted by VGluT signals. ****P < 0.0001, Chi-square test.
(**C**) Proportion of spines after image rotation in which Homer1 was contacted by the same VGluT signal as the original image.

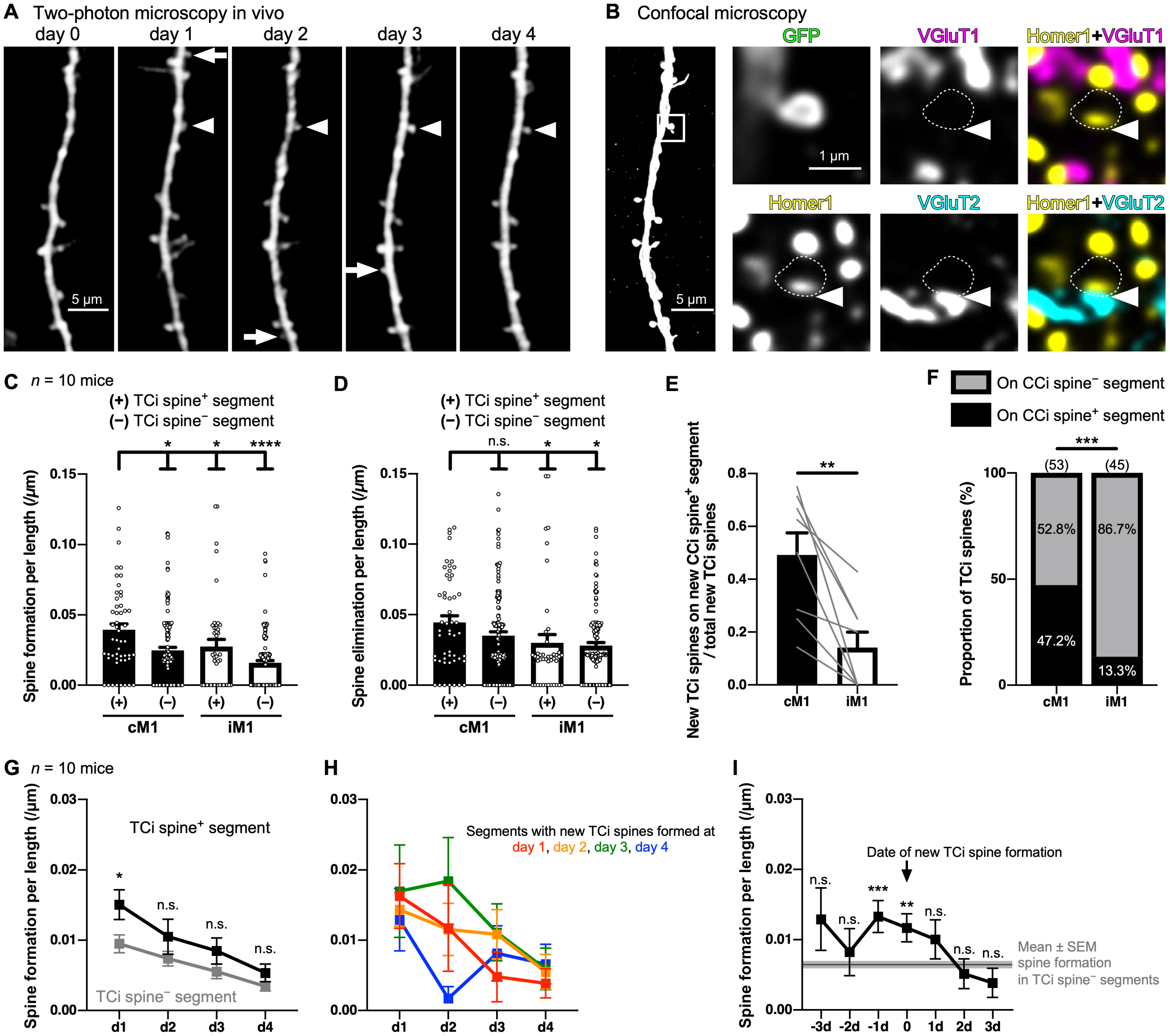

Fig. S6. New TCi spines are formed preferentially at the dendritic segments with high formation activity.

(**A**) Two-photon images during 4-day training. Arrowheads indicate a newly-formed spine persisting until day 4. Arrows indicate transient new spines eliminated by day 4.

(**B**) Confocal images of the new spine corresponding to (**A**). The new spine is innervated by a TC axonal bouton. Arrowheads indicate a putative synaptic contact site.

(**C**) Spine formation activity (total number of spines per length [µm] formed during 4-day training including transiently-formed spines) on the dendritic segments with and without new TCi spines observed at day 4 in bilateral M1s (cM1 with new TCi spines, 51.4 ± 1.2 µm [n = 51]; iM1 with new TCi spines, 51.4 ± 1.2 µm [n = 43]; cM1 without new TCi spines 51.2 ± 0.7 µm [n = 127], mean ± SEM in the region of interest). The formation activity on the segments with new TCi spines in cM1 is significantly higher than in iM1. The spine formation activity is also higher in the segments with TCi spines than those without TCi spines in cM1. $P < 0.0001$, Kruskal-Wallis test; cM1-TCi$^+$ vs. cM1-TCi$^-$, vs. iM1-TCi$^+$, and vs. iM1-TCi$^-$, P = 0.0108, 0.0485, and < 0.0001, respectively, Dunn's multiple comparisons test.

(**D**) Spine elimination activity (total number of spines per length [µm] eliminated during 4-day training including transiently-formed spines) on the dendritic segments with and without new TCi spines observed at day 4 in bilateral M1s (mean ± SEM). P = 0.0110, Kruskal-Wallis test; cM1-TCi^{+} vs. cM1-TCi^{-}, vs. iM1-TCi^{+}, and vs. iM1-TCi^{-}, P = 0.6910, 0.0211, and 0.0413, respectively, Dunn's multiple comparisons test.
(**E**) Frequency of new TCi spines on the segments with new CCi spines found at day 4 in bilateral M1s (mean ± SEM). Gray lines show data of individual mice. $W = -36$, P = 0.0078, two-tailed Wilcoxon matched-pairs signed rank test.
(**F**) Proportion of new TCi spines on the segments with or without new CCi spines. P = 0.0004, two-sided Fisher's exact test.
(**G**) Spine formation activities in new TCi spine^{+} and new TCi spine^{-} segments in cM1 between days 0 and 4. The data include new spines that were formed by day 4, regardless of whether they were observable at day 4 or pruned by day 4. Spine formation activity was significantly higher in new TCi spine^{+} segments than new TCi spine^{-} segments at day 1 (U = 2536, P = 0.0144, Mann Whitney test), whereas the differences from day 2 to day 4 were not statistically significant (U = 3048, P = 0.5262 at day 2; U = 2824, P = 0.1024 at day 3; U = 2900, P = 0.1495 at day 4).
(**H**) Spine formation activities in new TCi spine^{+} segments in cM1. Data of the segments with new TCi spines formed at each experimental day are displayed in different colors; mean ± SEM of spine formation activity in the segments with new TCi spines formed at day 1 (and sustained until day 4) is colored in red, and those at days 2, 3, and 4 are in orange, green and blue, respectively.
(**I**) The spine formation activities at each training day aligned to the date of new TCi spine generation. "-3d" means 3 days before the formation of new TCi spines; in case of the segments with new TCi spines formed at day 4, the data in "-3d" indicate the spine formation activity at day 1. The value at "0d" represents the number of newly-formed spines at the equivalent date of TCi spine generation in the same segment (the new TCi spine is excluded from the value). The gray horizontal line indicates mean ± SEM of the number of newly-formed spines per length on new TCi^{-} segments. The values at "-1d" and "0d" were significantly higher than the mean on new TCi^{-} segments, indicating that frequent spine formation occurs exactly at the same day of or the day before new TCi spine generation (U = 2317, P = 0.0694 at -3d; U = 5161, P = 0.8099 at -2d; U = 6447, P = 0.0003 at -1d; U = 10303, P = 0.0033 at 0d; U = 8867, P = 0.1977 at 1d; U = 7151, P = 0.5254 at 2d; U = 3443, P = 0.4359 at 3d; Mann Whitney test).
*P < 0.05, **P < 0.01, ***P < 0.001, ****P < 0.0001.

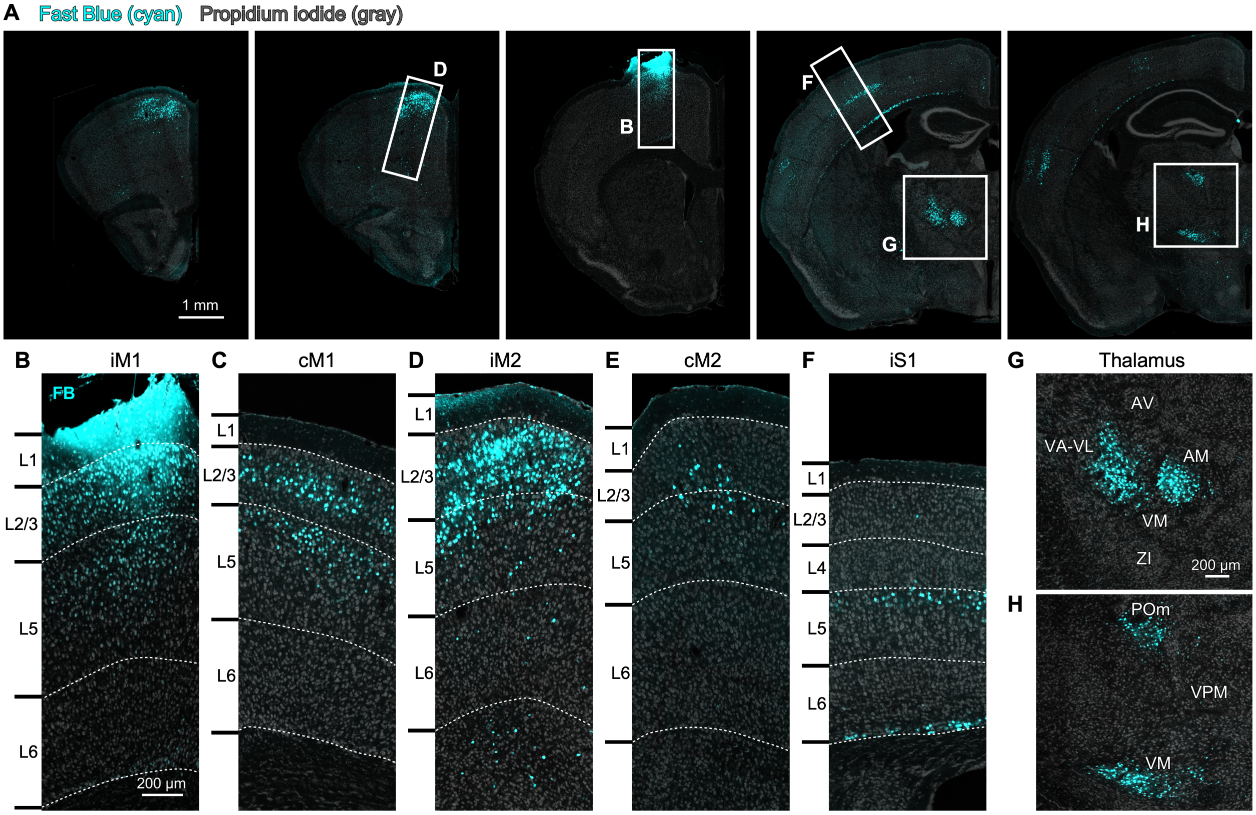

Fig. S7. Retrograde labeling of neurons that project to layer 1 of M1.

(**A**) Low-magnification images of coronally-sectioned brain tissues with Fast Blue labeling (cyan) and propidium iodide counterstaining (gray).

(**B**) Local immersion of Fast Blue (FB) into layer (L) 1 of M1.

(**C** to **F**) Cell bodies retrogradely labeled with Fast Blue in M1 contralateral to the Fast Blue-immersed hemisphere (cM1, **C**), the ipsilateral M2 (iM2, **D**), the contralateral M2 (cM2, **E**), the ipsilateral primary somatosensory cortex (iS1, **F**).

(**G** and **H**) Fast Blue-labeled cell bodies in the thalamic nuclei ipsilateral to the Fast Blue-immersed hemisphere. AM, anteromedial thalamic nucleus; AV, anteroventral thalamic nucleus; POm, posterior thalamic nucleus; VA-VL, ventral anterior-ventral lateral nuclear complex; VM, ventral medial nucleus; VPM, ventral posteromedial thalamic nucleus; ZI, zona incerta.

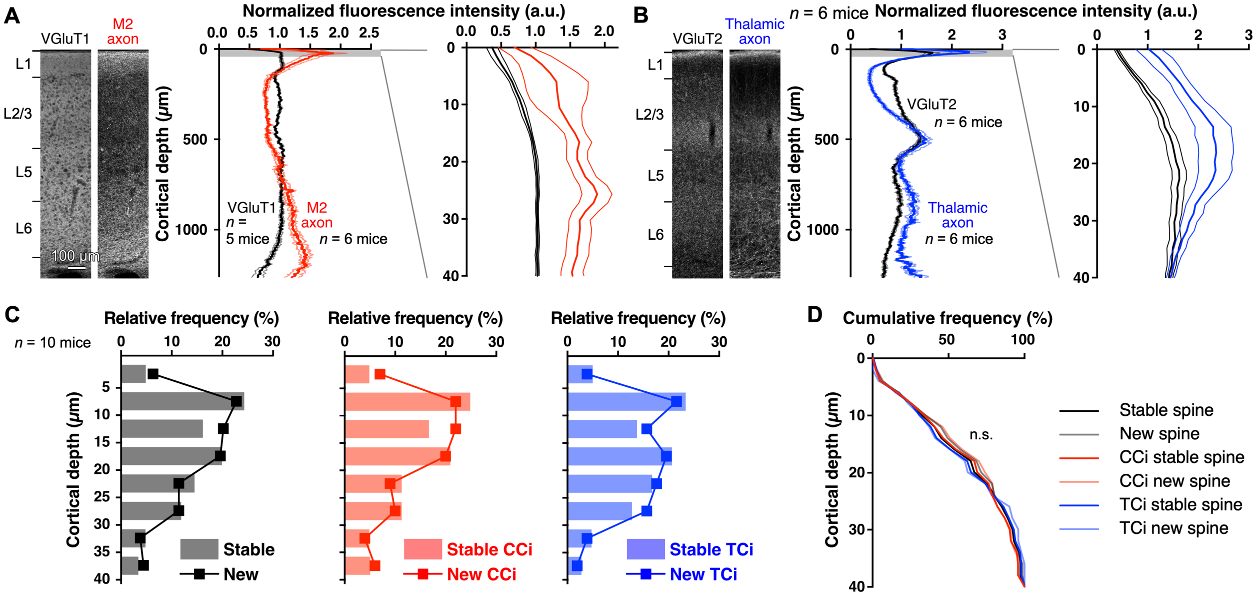

Fig. S8. Laminar distributions of observed CCi and TCi spines in M1.

(**A**) Laminar fluorescence-signal distributions of VGluT1 (mean ± SEM; n = 5 mice) and M2 axons (mean ± SEM; n = 6 mice) in M1. M2 axons were labeled with the injection of AAV-mCherry into M2 (see Fig. 3, D and E). Fluorescence intensities are normalized to the mean intensities between 0 and 1 mm of the cortical depth (1 a.u.). The laminar distributions within 40 µm from the pial surface are magnified in the right panel. Also see Fig. 3B. L, layer.

(**B**) Laminar fluorescence-signal distributions of VGluT2 (mean ± SEM; n = 6 mice) and thalamic axons (mean ± SEM; n = 6 mice) in M1. Thalamic axons were labeled with the injection of AAV-mCherry into the motor thalamic nuclei (see Fig. 3, D and F). Fluorescence intensities are normalized to the mean intensities between 0 and 1 mm of the cortical depth (1 a.u.). The laminar distributions within 40 µm from the pial surface are magnified in the right panel. Also see Fig. 3C.

(**C**) Distributions of stable and new spines observed in this study. The dendrites within 40 µm from the cortical surface were analyzed. The observed spines are largely distributed in the upper part due to the arborization pattern of the apical tufts. Both stable and new spines observed show similar vertical distributions.

(**D**) Cumulative frequencies of analyzed stable and new spines along the cortical depth. The distributions were not significantly different compared with the distribution of all stable spines (All new spines, D = 0.04453, P = 0.9361; stable CCi spines, D = 0.03079, P = 0.7836; new CCi spines, D = 0.06934, P = 0.7533; stable TCi spines, D = 0.03219, P = 0.5241; new TCi spines, D = 0.05963, P = 0.9946; Kolmogorov-Smirnov test).

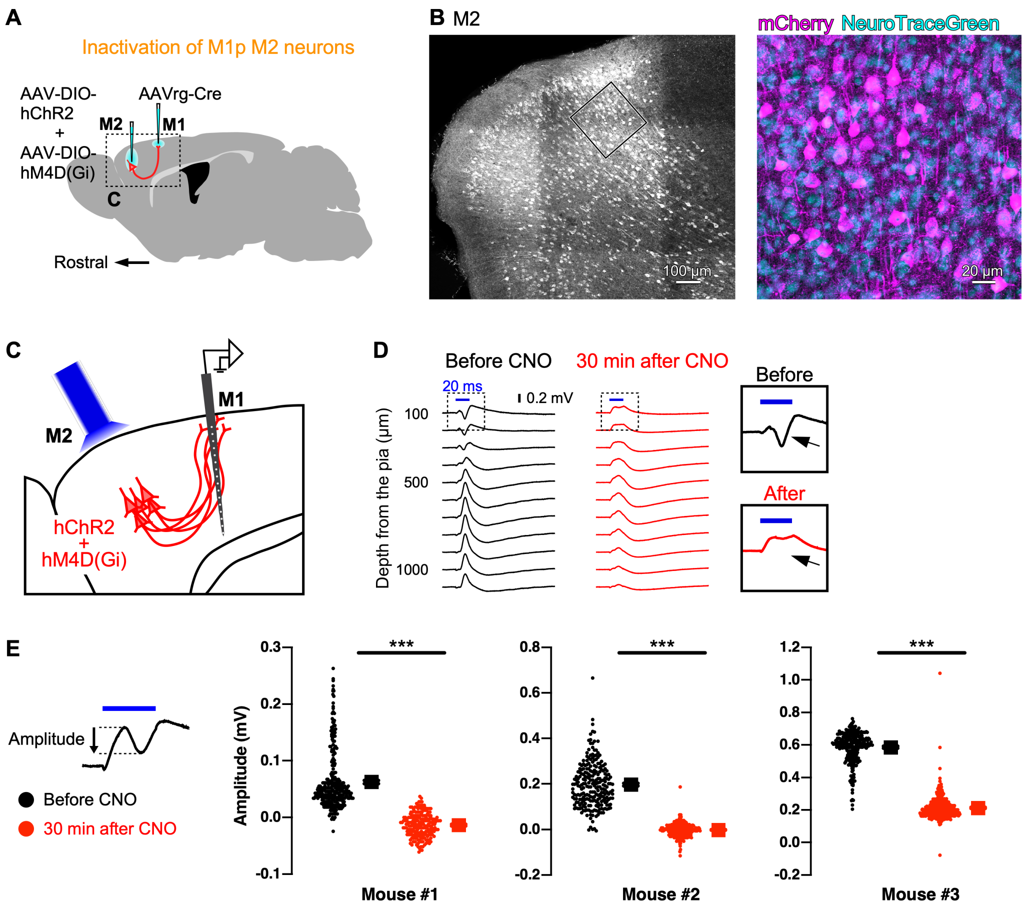

Fig. S9. Effect of chemogenetic silencing of M1p M2 neurons on postsynaptic activity.

(**A**) Virus injection for optogenetic activation with chemogenetic silencing of M1p M2 neurons. A mixture of AAV-DIO-hChR2 and AAV-DIO-hM4D(Gi) was injected into M2, combined with injection of AAVrg-Cre into M1.

(**B**) Confocal image of hM4D(Gi)-expressing cells in M2. AAV-infected M2 neurons also express mCherry with which hM4D(Gi) is tagged (magenta), counterstained with Green Fluorescent Nissl staining (NeuroTrace Green, pseudo-colored cyan). hM4D(Gi) was expressed in 21.3 ± 2.2% (825/3,957 cells), 15.5 ± 1.7% (630/4,343 cells) and 10.4 ± 1.7% (450/4,606 cells) of L2/3, L5 and L6 neurons, respectively, at the injection sites (n = 11 mice; mean ± SEM).

(**C**) Recording of M1 response evoked by M2 neuron stimulation 4 weeks after the injection of AAVs. A 16-channel probe was inserted into M1, while M2 neurons were excited by blue-light illumination.

(**D**) Field potentials evoked by optogenetic stimulation of M2 neurons before and 30 min after systemic CNO injection. Each trace represents the average over 243 measurements. Blue line indicates the duration of blue light (20 ms). Response at the superficial layer in M1 to optogenetic stimulation of M2 was markedly reduced by CNO administration.

(**E**) Evoked amplitudes at 100 µm below the pial surface before and after CNO injection (mean ± SEM). The amplitudes of evoked potentials were measured between peaks (*81*). All animals showed significant reduction of the amplitudes (n = 3 mice, 213–393 measurements from each recording; U = 1413, 680 and 916.5, all $P < 0.0001$, two-tailed Mann Whitney test). ***$P <$ 0.001.

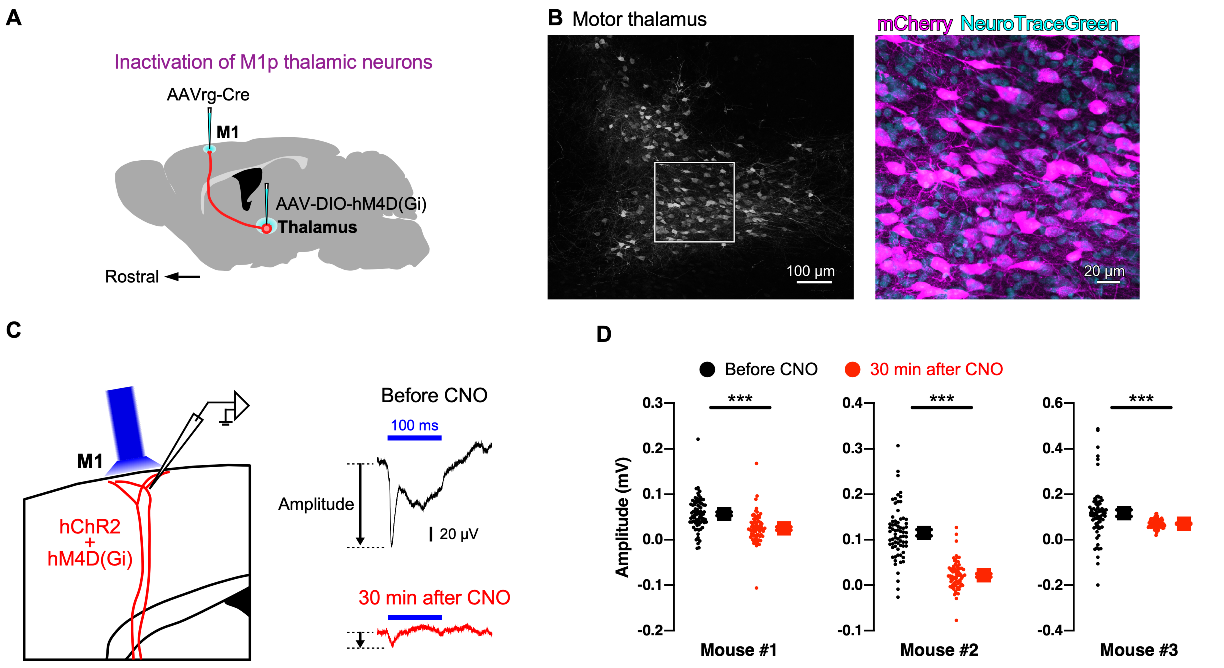

Fig. S10. Effect of chemogenetic silencing of M1p thalamic neurons on postsynaptic activity in M1.

(**A**) Virus injection for hM4D(Gi) expression in M1p thalamic neurons.
(**B**) Confocal image of hM4D(Gi)-expressing cells in the motor thalamic nuclei. AAV-infected thalamic neurons co-express mCherry (pseudo-colored magenta), counterstained with Green Fluorescent Nissl staining (NeuroTrace Green, pseudo-colored cyan). hM4D(Gi) was expressed in 35.6 ± 2.6% (827/2,377 cells) of thalamic neurons at the injection sites (n = 12 mice; mean ± SEM).
(**C**) (Left) Recording of M1 response evoked by thalamic axon stimulation 4 weeks after the injection of AAVs. A mixture of AAV-DIO-hChR2 and AAV-DIO-hM4D(Gi) was injected into the thalamus, combined with injection of AAVrg-Cre into M1 for optogenetic activation with chemogenetic silencing of M1p thalamic neurons. A glass pipette was inserted into M1, while thalamic axons were excited by blue-light illumination. (Right) Field potentials evoked by optogenetic stimulation of thalamic axons in M1 before (black trace) and 30 min after systemic CNO injection (red trace). Traces represent the average over 70 measurements. Blue line indicates the duration of blue light (100 ms). Response to optogenetic stimulation of thalamic axons was reduced by CNO administration.
(**D**) Evoked amplitudes at 20–50 µm below the pial surface before and after CNO injection (mean ± SEM). The amplitudes of evoked potentials were measured between peaks. All animals showed significant reduction of the amplitudes (n = 3 mice, 70–80 measurements from each recording; U = 1380, 344 and 1331, all $P < 0.0001$, two-tailed Mann Whitney test). ***$P <$ 0.001.

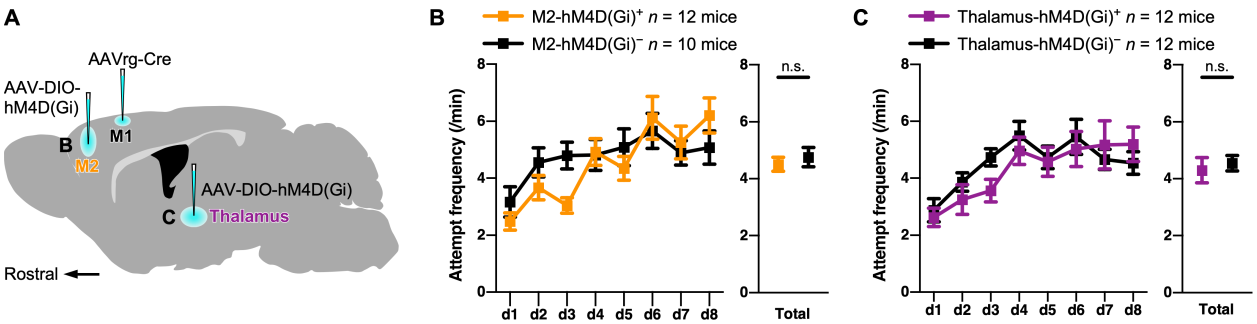

Fig. S11. Effect of chemogenetic silencing of M1p M2 or M1p thalamic neurons on reaching behavior.

(**A**) Virus injection for chemogenetic silencing of M1p M2 or M1p thalamic neurons.
(**B** and **C**) Frequencies (/min) of reaching attempts with chemogenetic inactivation of presynaptic activity (mean ± SEM). Neither chemogenetic silencing of M1p M2 (**B**) nor thalamic (**C**) neurons significantly affected attempt frequencies (U = 45 and 67, P = 0.3463 and 0.7987, respectively; two-tailed Mann Whitney test).

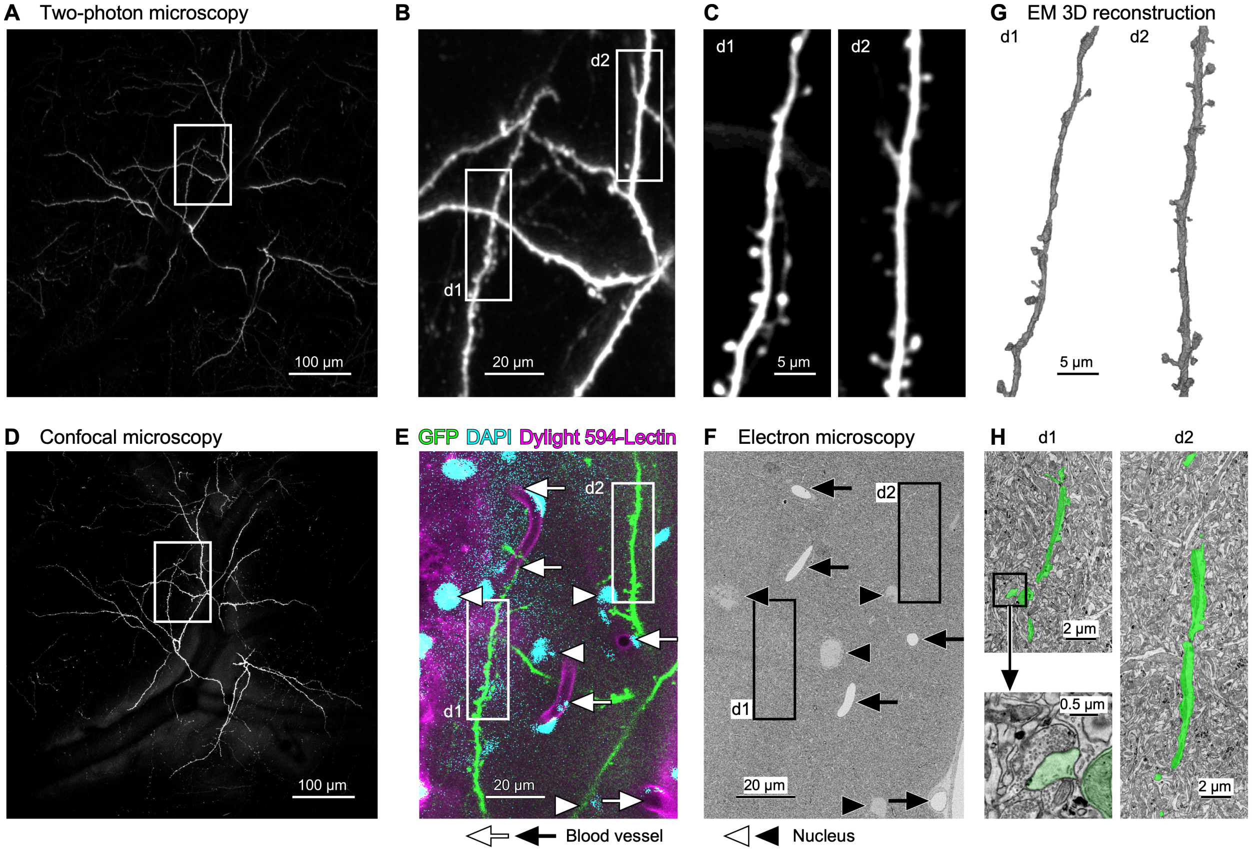

Fig. S12. Correlated two-photon, confocal and electron microscopy.

(**A**) A two-photon low-magnification image in vivo.
(**B** and **C**) Two-photon high-magnification images. (**B**) corresponds to the rectangle in (**A**), and (**C**) corresponds to the rectangles in (**B**).
(**D**) A confocal image of the apical tufts corresponding to (**A**).
(**E**) A confocal image of GFP-labeled dendrites with DAPI and DyLight 594-Lectin counterstaining. GFP-native fluorescence (dendrites), nuclei and capillaries are shown in pseudocolored green, cyan and magenta, respectively. Arrows and arrowheads indicate capillaries and nuclei, respectively.
(**F**) An EM image with ATUM-SEM. Capillaries and nuclei can be observed at the location corresponding to (**E**). Such landmarks allow reidentification of the dendritic segments observed in two-photon and confocal images.
(**G**) Reconstructed dendritic segments observed in (**C**).
(**H**) High-magnification EM images in (**F**). Dendritic segments are shown in green.

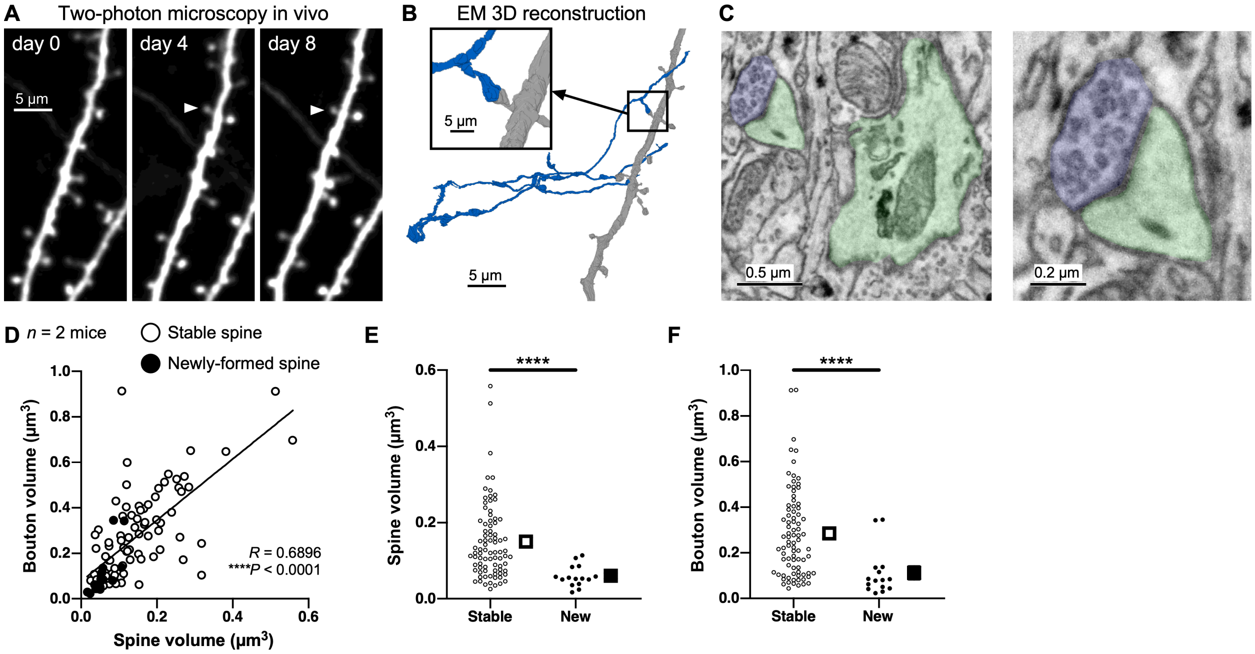

Fig. S13. New spines are small in the head and presynaptic bouton volume.

(**A**) Two-photon images in vivo during 8 days of training. Arrowheads indicate a persistently-formed new spine.

(**B**) EM 3D reconstruction of the corresponding dendrite (gray) to (**A**). An arrowhead indicates a synaptic contact site of a persistently-formed spine with an axon fiber (blue).

(**C**) EM images of the synaptic input site indicated by the arrowhead in (**B**). A bouton is shaded in blue, while a dendrite is in green.

(**D**) Size correlation between pairwise spines and boutons. $R = 0.6896$, $P < 0.0001$, Pearson correlation coefficient.

(**E** and **F**) Size comparisons of spines (**E**) or boutons (**F**) forming stable and new spines (mean ± SEM). Both $P < 0.0001$.

****$P < 0.0001$.

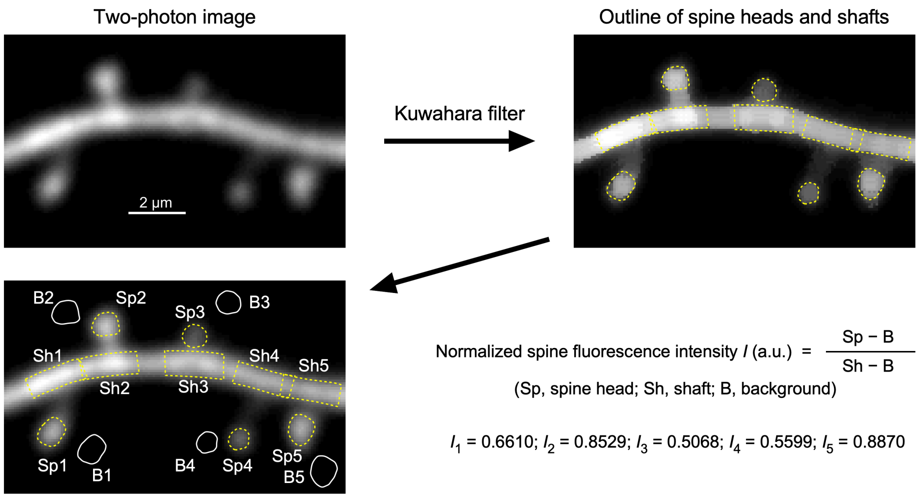

Fig. S14. Outline determination of spines and shafts for measurement of fluorescence intensity.

A two-photon image processed with Kuwahara filter (sampling window width is 5). The spine and shaft edges are outlined in dotted lines (yellow). Spine and shaft intensities were measured in single planes with the maximum intensities among all optical sections. Background intensities were measured near the spine head, with the equivalent spine sizes.

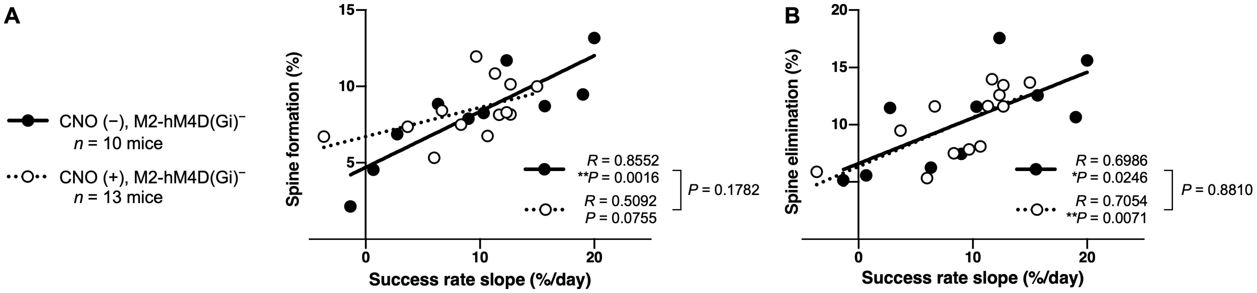

Fig. S15. Effect of CNO application on spine dynamics with or without hM4D(Gi) expression.

(**A**) Correlations between success rate slopes and spine formation rates of individual mice with or without systemic CNO application. Filled circles show spine formation rates and success rate slopes of individual mice without either CNO application or AAV injection, same as Fig. 1E. Open circles represent individual mice with systemic CNO application (and with injection of AAVre-Cre into M1 and AAV-DIO-mCherry into M2 or the thalamus) as illustrated in Fig. 4, C and F. The linear regression lines were not significantly different between the two groups (*P* = 0.1782, ANCOVA), indicating that systemic CNO application does not affect spine formation in cM1.

(**B**) Correlations between success rate slopes and spine elimination rates of individual mice with or without systemic CNO application. Filled circles show spine elimination rates and success rate slopes of individual mice without either CNO application or AAV injection, same as Fig. 1G. Open circles represent individual mice with systemic CNO application and with AAV injection (hM4D(Gi)$^{-}$). The difference of the linear regression lines between the two groups was statistically insignificant (*P* = 0.8810, ANCOVA), indicating that systemic CNO application does not affect spine elimination as well as spine formation.

Supplementary Movie Captions

Movie S1. Single-seed reaching task.
The mouse becomes proficient at seed grasping from day 1 (left) to day 4 (right).

Movie S2. Correlated confocal and electron microscopy.
(Left) A fixed brain section is stained in fluorescence with DAPI (cyan) and DyLight 594-labeld Lycopersicon Esculentum (Tomato) Lectin (magenta). DAPI and DyLight 594 visualize cell nuclei and blood vessels, respectively. (Right) EM image stacks captured with ATUM-SEM. Cell nuclei and blood vessels are observable at the location almost identical to the confocal image. The dendritic segment in the square is three-dimensionally reconstructed.
